# Palaeoproteomics confirm earliest domesticated sheep in southern Africa ca. 2000 BP

**DOI:** 10.1038/s41598-021-85756-8

**Published:** 2021-03-23

**Authors:** Ashley N. Coutu, Alberto J. Taurozzi, Meaghan Mackie, Theis Zetner Trolle Jensen, Matthew J. Collins, Judith Sealy

**Affiliations:** 1 Pitt Rivers Museum, University of Oxford, Oxford, OX1 3PP UK; 2 BioArCh, University of York, York, YO10 5DD UK; 3 Section for Evolutionary Genomics, GLOBE Institute, Faculty of Health and Medical Science, University of Copenhagen, Øster Farimagsgade 5, 1353 Copenhagen, Denmark; 4 Novo Nordisk Foundation Center for Protein Research, University of Copenhagen, Blegdamsvej 3b, 2200 Copenhagen N, Denmark; 5 McDonald Institute for Archaeological Research, University of Cambridge, West Tower, Downing St, Cambridge, CB2 3ER UK; 6 Department of Archaeology, University of Cape Town, Private Bag X3, Rondebosch, 7701 South Africa

**Keywords:** Proteomic analysis, Archaeology

## Abstract

We used palaeoproteomics and peptide mass fingerprinting to obtain secure species identifications of key specimens of early domesticated fauna from South Africa, dating to ca. 2000 BP. It can be difficult to distinguish fragmentary remains of early domesticates (sheep) from similar-sized local wild bovids (grey duiker, grey rhebok, springbok—southern Africa lacks wild sheep) based on morphology alone. Our analysis revealed a Zooarchaeology by Mass Spectrometry (ZooMS) marker (*m/z* 1532) present in wild bovids and we demonstrate through LC–MS/MS that it is capable of discriminating between wild bovids and caprine domesticates. We confirm that the Spoegrivier specimen dated to 2105 ± 65 BP is indeed a sheep. This is the earliest directly dated evidence of domesticated animals in southern Africa. As well as the traditional method of analysing bone fragments, we show the utility of minimally destructive sampling methods such as PVC eraser and polishing films for successful ZooMS identification. We also show that collagen extracted more than 25 years ago for the purpose of radiocarbon dating can yield successful ZooMS identification. Our study demonstrates the importance of developing appropriate regional frameworks of comparison for future research using ZooMS as a method of biomolecular species identification.

## Introduction

**Figure 1:**
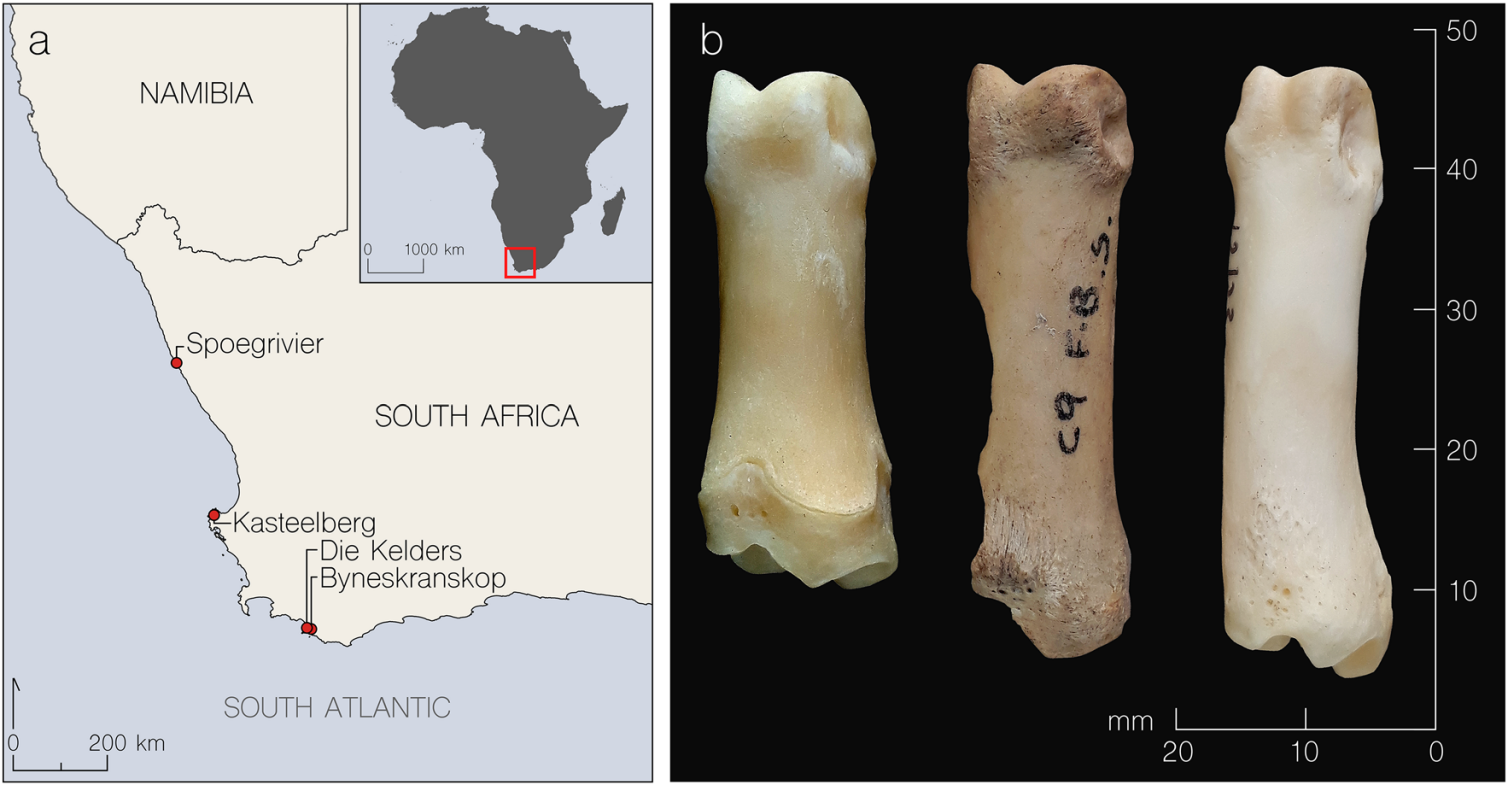
Location of archaeological sites and image of oldest sample investigated (P4859C) (**a**) Map showing locations of archaeological sites that yielded specimens analysed in this study. (**b**) First phalanx from Spoegrivier dated to 2105 ± 65 BP (centre) compared with modern sheep (left) and springbok (right).

The transition from hunting and gathering to food production is one of the most important processes in human history. In parts of sub-Saharan Africa, domesticated animals appeared earlier than domesticated plants^1^. Domesticated sheep (*Ovis aries*) were introduced into North Africa from western Asia, spreading to eastern Africa by about 5000 BP and then southwards. There are no wild sheep in southern Africa, therefore the earliest animals were undoubtedly imported and were probably the earliest domesticates in southern Africa^2,3^. The timing is hotly debated. Domesticated sheep have long been thought to have reached southernmost Africa ca. 2000 BP^4,5^, but the initial studies relied on dating by association—at that time, only ‘conventional’ radiocarbon dating was possible, necessitating large samples. Most dates were derived from wood charcoal, and considered to apply also to faunal and other assemblages from the same stratigraphic levels. Subsequently, direct AMS radiocarbon dating of supposedly early sheep bones showed that many were actually younger than the layers in which they were found^6–8^, although a few did in fact date to ca. 2000 BP. A sheep astragalus from Toteng 1 in northern Botswana is directly dated by AMS to 2020 ± 40 BP (Beta-186669)^9,10^. Part of a sheep mandible and a calcaneum from Blombos Cave, on the south coast, are similarly dated to 1960 ± 50 BP and 1880 ± 55 BP (OxA-4543 and OxA-4544 respectively)^11^. The oldest date reported for cattle bone in southern Africa is also from Toteng: 2070 ± 40 (Beta-190488)^9,10^. One sheep phalanx from the site of Spoegrivier in Namaqualand (Fig. 1) is directly AMS dated to 2105 ± 65 BP (OxA-3862)^7^, which calibrates at two sigma to 351-308 BC [4.8% probability] or 197 BC- 110 AD [90.7% probability], using OxCal 4.4 and the SHCal20 calibration curve^12,13^). At present, this is the oldest secure date for domesticated animals in southern Africa. Taken as a whole, the chronology based on individually dated sheep or cattle bones supports the introduction of domesticated animals into southern Africa shortly before 2000 BP^14^. The number of specimens is small, so each example is critically important.

There remains a question as to whether the species identifications of these specimens are secure. All those mentioned above have been identified only by their morphology, and confusion with several similar-sized species of wild bovids is possible, as highlighted by the mismatch between morphological and ancient DNA (aDNA) identifications of putative early domesticates at the site of Blydefontein in central South Africa^15–19^, at Sehonghong in Lesotho^20–22^ and elsewhere^23^. Several teeth from Leopard’s Cave in Namibia, dated to just over 2000 BP and originally thought to be from domesticated caprines (sheep and/or goat), have since been shown by Le Meillour et al. (2020) to derive from wild springbok^24^. This study, like Le Meillour et al. (2020), uses proteomic analysis to distinguish species based on species-specific protein sequences. The archaeological bones included in this study were originally identified as sheep by faunal expert Richard Klein, based on morphological criteria. These specimens were selected jointly by Richard Klein, Royden Yates and Judith Sealy for AMS radiocarbon dating to begin to establish a more secure chronology for the introduction of domesticated stock into southern Africa based on direct AMS dates rather than dating by association^7,8^. A key criterion for selection was the choice of skeletal elements most clearly diagnostic of sheep, as opposed to wild species. These specimens are unlikely to be goats, since domesticated goats have not been recorded from precolonial times in south-western South Africa, although they were present in the northern and eastern parts of the country^5,25^. There remains, however, a possibility that the morphological identifications as sheep may be wrong. In recent years, as biomolecular methods of species identification have developed, the expectation has arisen that these should be used to cross-check morphological identification of key early specimens of domesticates that form the cornerstones of our chronologies for the introduction of food production in different parts of the world^17,26–31^.

Biomolecular approaches to species identification augment morphological species assignment, especially when remains are fragmentary, poorly preserved and/or from non-diagnostic skeletal elements. A number of recent studies have utilised ancient protein (or palaeoproteomic) techniques to identify archaeological bone and ivory remains in sub-Saharan Africa. Le Meillour et al. developed a protocol for the identification of degraded bone fragments and applied it to the identification of remains from Toteng^32^ and from Leopard’s Cave in Namibia^24^. Zooarchaeology by Mass Spectrometry (ZooMS) has been used to identify animal species from which bone was selected to fashion into artefacts^33^, to distinguish East African sheep and goats^2^, to identify Asian fauna moving into eastern Africa^30^, and to investigate early farmers’ choice of elephant or hippo ivory in south-eastern Africa^34^. Proteomic techniques have also been used to identify the oldest authenticated proteins ever found, from eggshell at the site of Laetoli, Tanzania, 3.8 Ma^35^.

ZooMS is one of the most widely used and cost-effective ancient protein analytical strategies and is now well-established as a method of identifying hominid and zooarchaeological remains^36^. It uses matrix-assisted laser desorption/ionization time-of-flight (MALDI-TOF) mass spectrometry to obtain characteristic profiles of protein fragments (or peptides), typically from collagen, extracted from archaeological bone. Different taxa have slight variations in the amino acid sequences of their collagen so the resulting peptide mass ‘fingerprints’ provide markers that in many cases allow robust and objective species identification. New markers identified by ZooMS can subsequently be validated by Liquid Chromatography Tandem Mass Spectrometry (LC–MS/MS), the ‘gold standard’ of proteomic analysis. This powerful technique allows direct amino acid sequencing of proteomes and proteins, enabling verification of phylogenetically relevant sites. LC–MS/MS is not based simply on the total mass of the peptide, like ZooMS, but also on secondary fragmentation of the peptide to interrogate each amino acid. ZooMS and other palaeoproteomic methods also offer some advantages compared with aDNA. Collagen is a robust biomolecule, which has been shown to preserve longer than aDNA^37^. In addition, the amount of sample required is typically smaller than that needed for aDNA analysis, involving less destruction of precious specimens, and some methods of sampling, e.g. eraser rubbings, are effectively non-destructive^38–40^.

ZooMS has proven successful in distinguishing sheep from morphologically similar species, especially goats, in various parts of the world^2,28,41,42^. Successful application depends on the ZooMS fingerprint of sheep showing clear differences from those of local wild fauna and other domesticates. This is particularly important in Africa, home to many species of wild bovids that have not yet been fully investigated using proteomic approaches. In this paper, we focus on south-western Africa, where these techniques have been underutilised, comparing 8 archaeological specimens morphologically identified as sheep with the three species of similar-sized wild bovids with which sheep bones are most likely to be confused (Table 1). These are *Antidorcas marsupialis* (springbok), *Pelea capreolus* (grey rhebuck) and *Sylvicapra grimmia* (grey or common duiker). This last is rather smaller than sheep, so confusion is less likely, but we include it here for completeness. In other parts of Africa there are additional wild species (e.g. *Aepyceros melampus*, impala) that may be confused with sheep, but impala do not occur in our study area so are not included in our reference samples.

**Table 1:** Species, provenance, and dates for modern reference and archaeological samples in this study.

Sample ID	Site	Accession number/layer details	Species/skeletal element	Species common name/radiocarbon lab number	Date	Sample type
**Modern reference samples**						
A	Karoo	91/3	*Antidorcas marsupialis*	Springbok	1991	B
B	Richmond, Karoo	90/13	*Antidorcas marsupialis*	Springbok	1990	B
C	Namaqua National Park		*Antidorcas marsupialis*	Springbok	2014	B
D	Western Cape		*Pelea capreolus*	Grey rhebok	1980	B
F	Pongola	11/76	*Ovis aries*	Zulu sheep	2010	B
G	Upington	9/6	*Ovis aries*	Namaqua Afrikaner sheep	2009	B
I	Calvinia		*Ovis aries*	Damara sheep		B
J	Carnarvon	15/99	*Ovis aries*	Namaqua Afrikaner sheep	2015	B
K	Thohoyandou	12/18	*Capra aegagrus hircus*	Mbuzi veld goat	2012	B
L	Stutterheim, Eastern Cape	9/3	*Capra aegagrus hircus*	Xhosa lob-ear goat	2009	B
M	Western Cape	13/314	*Sylvicapra grimmia*	Grey duiker	2013	B
N	Stellenbosch	15/11	*Sylvicapra grimmia*	Grey duiker	2015	B
**Archaeological samples**						
P4859	Spoegrivier	FBS C9	Right first phalanx	OxA-3862	2105 ± 65 BP	B
P4859C	Spoegrivier	FBS C9	Right first phalanx	OxA-3862	2105 ± 65 BP	C
P4863	Kasteelberg A	20–30 A20	Thoracic vertebra, juvenile	OxA-3864	1630 ± 60 BP	B
P4864	Kasteelberg A	20–30 A20	Distal metapodial, adult	OxA-3865	1430 ± 55 BP	B
P4772	Die Kelders	Layer 7 B5	Second phalanx, juvenile	OxA-3860	1325 ± 60 BP	C
P4773	Die Kelders	Layer 7 AA8	Maxillary fragment with P2	OxA-3861	1290 ± 60 BP	C
P4862	Byneskranskop 1	Layer 1 O30	Right mandibular condyle (small)	OxA-3863	1370 ± 60 BP	B
P4861	Byneskranskop 1	Layer 1 O30	Right mandibular condyle (large)	N/A	N/A	B
P4865	Kasteelberg A	40–50 21a	Maxillary fragment with juvenile upper left DP4	N/A	N/A	B

Radiocarbon dates from Sealy and Yates (1994)^7^. Sample type “B” is bone, “C” is collagen extracted by the Oxford Radiocarbon Accelerator Unit in 1992-3 for AMS dating. Remains of those extracts were returned to us for this study. Date indicates time of death for the modern reference samples, and radiocarbon dates for the archaeological ones.

The aims of this research were, therefore (1) to identify ZooMS peptide markers capable of distinguishing sheep from wild south-west African bovids of similar size and morphology, (2) to search for these markers in faunal remains believed to be those of domesticated sheep, especially the earliest directly dated sheep specimen in southern Africa, from Spoegrivier, and (3) to assess the viability of minimally destructive collagen extraction for archaeological samples from these contexts. To achieve these aims we first utilised ZooMS and spectral comparison of modern reference material to identify candidate markers. We subsequently validated these candidates using LC–MS/MS.

## Results

### ZooMS and LC–MS/MS—modern reference samples

The modern domesticated sheep and goat samples from South Africa analysed here have collagen peptide mass fingerprints identical to those previously published for domestic sheep and goat in other parts of the African continent, and the world^2,27,28^. Specifically, these include the presence of distinguishing peptides at *m/z* 3033 in sheep and *m/z* 3093 in goat. The peptide mass fingerprint for grey rhebok differs from domesticated sheep and goat by the presence of peaks at *m/z* 1150, *m/z* 1166, *m/z* 1532 and *m/z* 1550. The peptide mass fingerprint for grey duiker differs from domesticated sheep and goat by the presence of peaks at *m/z* 1192, *m/z* 1208, *m/z* 1532. The peptide mass fingerprint for springbok differs from domesticated sheep and goat with the presence of peaks at *m/z* 1532, *m/z* 1550 and *m/z* 2553 (Supplementary Fig. S1 and Table S1).

**Figure 2:**
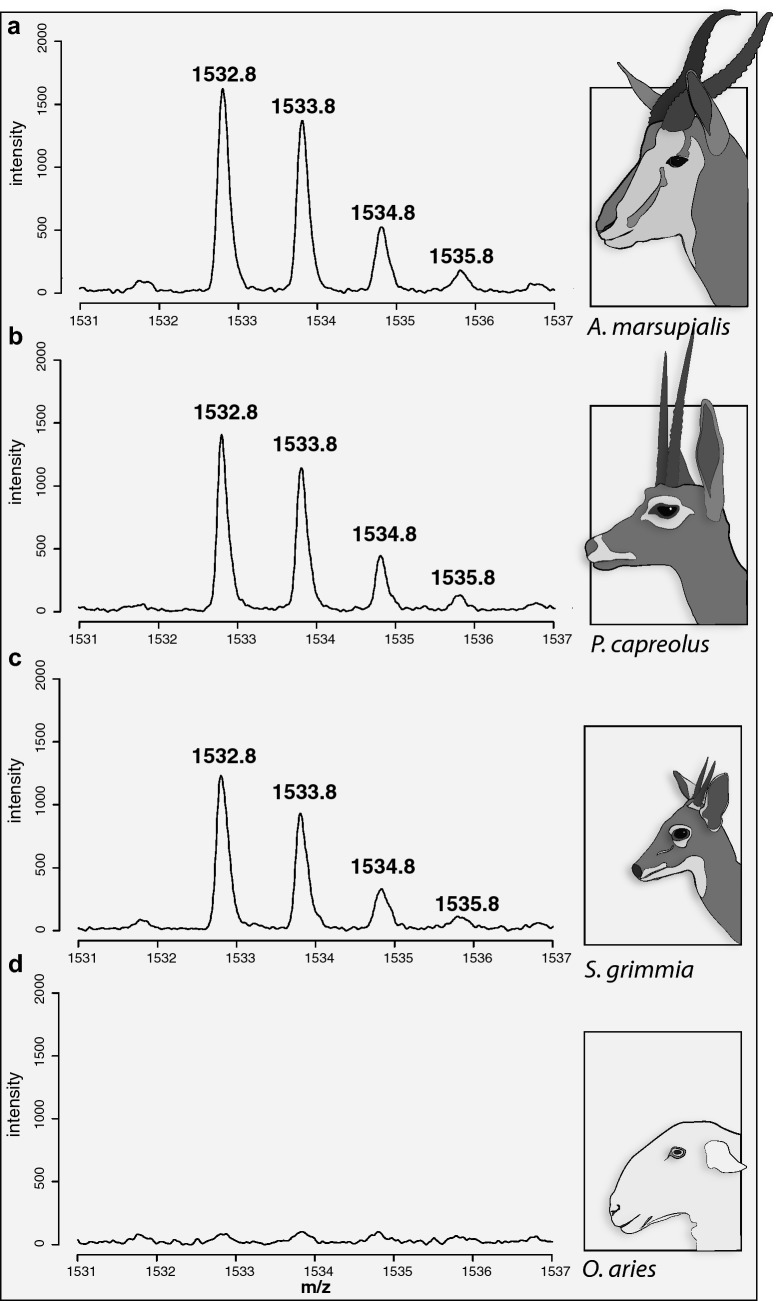
Presence and absence of species-specific mass ions in four ZooMS spectra. (**a**–**c**) Reference spectra of modern springbok, grey rhebok and common duiker show the presence of peptide with a *m/z* of 1532.8 and related ions. (**d**) Modern sheep reference spectrum showing the absence of the peptide with a *m/z* of 1532.8.

**Figure 3:**
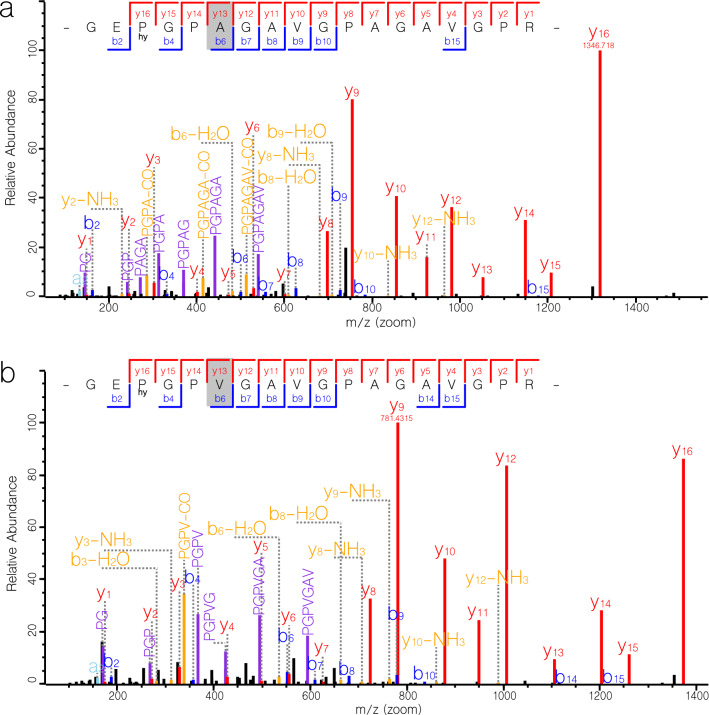
LC–MS/MS spectra from (**a**) modern reference springbok A and (**b**) modern reference sheep G. Grayed amino acids represent SAPs used to differentiate between the two species.

The peaks at *m/z* 1550 and *m/z* 2553 did not always appear or show high intensity in the modern wild bovid spectra. It is a well-reported phenomenon that certain peptides are ‘poor flyers’ in MALDI-TOF^43^. We therefore decided to investigate only the peak at *m/z* 1532 further, since it was present in all three wild bovids (Fig. 2). We used LC–MS/MS of reference samples A (springbok), D (grey rhebok), G (Namaqua Afrikaner sheep), and M (grey duiker) to validate this candidate marker for discriminating domesticated sheep from similar size wild bovids in this region. LC–MS/MS confirmed the presence of this marker (GEPGP**A**GAVGPAGAVGPR (COL1A2 position 889–906 (from start of triple helix, see Brown et al. 2020^44^), underline is hydroxyproline) in all three wild bovids, but not in the modern sheep where the corresponding peptide was GEPGP**V**GAVGPAGAVGPR (Fig. 3; Supplementary Fig. S2). We were unable to detect a peak corresponding to this sequence (*m/z* 1571 (single hydroxyproline residue) or *m/z* 1555 (proline only)) in our ZooMS data for the reference sheep sample, despite its presence in the LC–MS/MS data. According to the BLASTp search, this peptide is specific to caprines (sheep and goat). The recreation of the springbok sequence (see Supplementary material and supplementary Table S2) also showed various single amino acid polymorphisms (SAPs) present between springbok and sheep that could be used to distinguish them by proteomics.

### ZooMS and LC–MS/MS—archaeological samples

Examination of the ZooMS spectra of the eight archaeological samples showed sheep-specific markers in all samples except Byneskranskop P4862, which had little protein and did not produce good enough spectra to enable identification (supplementary Table S1). All lack the *m/z* 1532 marker characteristic of wild bovids studied here. In addition, all have the marker at *m/z* 3033 seen in sheep and lack the marker at *m/z* 3093 seen in goats. Where both solution and minimally destructive (eraser or film) extraction methods were used, identifications were consistent (Table 2). The solution method was the most effective for species identification (23/24 samples were successfully assigned). All three minimally destructive methods had a success rate of > 50%, however aluminum film had the greatest success rate and PVC eraser had the lowest success rate (PVC eraser 6/11; aluminum film 9/11; diamond film 8/11). Of the six archaeological samples tested using the PVC eraser and the polishing films, only one (Byneskranskop P4862, mentioned above) failed to provide spectra good enough for identification; that sample also yielded insufficient protein for the solution method. The ZooMS identifications are thus consistent with the morphological identifications, indicating that the seven bone fragments from the archaeological sites of Spoegrivier, Kasteelberg A, Die Kelders and Byneskranskop 1 are sheep.

**Table 2:** Species identification of modern reference and archaeological samples using different extraction methods.

Sample ID	Extraction methods			
	Eraser	Aluminum film (15 μm)	Diamond film (3 μm)	Bone fragments
**Modern reference samples**				
A	Springbok	Springbok	Springbok	Springbok
B	Springbok	Springbok	Springbok	Springbok
C	Springbok	Springbok	Springbok	Springbok
D	X	X	Grey rhebok	Grey rhebok
F	X	Sheep	Sheep	Sheep
G	N/A	N/A	N/A	Sheep
I	N/A	N/A	N/A	Sheep
J	N/A	N/A	N/A	Sheep
K	N/A	N/A	N/A	Goat
L	N/A	N/A	N/A	Goat
M	N/A	N/A	N/A	Grey duiker
N	N/A	N/A	N/A	Grey duiker
**Archaeological samples**				
P4859 Spoegrivier	X	Sheep	Sheep	Sheep
P4859C Spoegrivier	N/A	N/A	N/A	Sheep
P4863 Kasteelberg A	Sheep	Sheep	Sheep	Sheep
P4864 Kasteelberg A	Sheep	Sheep	Sheep	Sheep
P4772 Die Kelders	N/A	N/A	N/A	Sheep
P4773 Die Kelders	N/A	N/A	N/A	Sheep
P4862 Byneskranskop 1	X	X	X	No ID
P4861 Byneskranskop 1	Sheep	Sheep	X	Sheep
P4865 Kasteelberg A	X	Sheep	X	Sheep

X indicates that minimally destructive sampling (PVC eraser/polishing film) was attempted but failed to produce spectra good enough for identification, while N/A indicates that minimally destructive sampling was not attempted.

**Figure 4:**
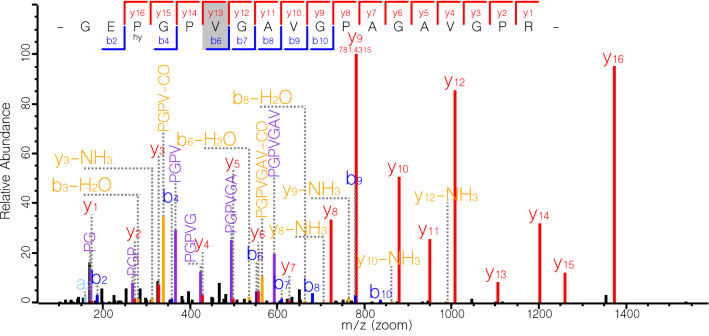
LC–MS/MS spectra for caprine specific peptide GEPGPVGAVGPAGAVGPR in sample P4859C from Spoegrivier, South Africa, morphologically assigned to sheep. The corresponding peptide for springbok, grey rhebok, grey duiker was not detected. Hy on y16 indicates the presence of hydroxyproline, greyed out V is indicative of species-specific amino acid.

In order to further validate the ZooMS markers seen in the spectra of the archaeological samples, collagen from the oldest sample from the site of Spoegrivier, P4859C, was also analysed by LC–MS/MS to allow for direct protein sequencing. The presence of the caprine version of the marker identified by ZooMS further validated the morphological identification as sheep (Fig. 4). In addition, when searched against the whole sheep proteome, this sample contained sheep-specific peptides for four additional proteins (alpha-2-HS-glycoprotein, collagen V, vitronectin, and a protein with homology to prothrombin), as well as three other caprine-specific proteins (vitamin K-dependent protein S, collagen VI, and perlecan) (Supplementary Tables S3 and S4). While the sequences of these proteins are not known for the three wild bovids considered here, these proteins are likely more taxonomically varied than collagen, which is highly conserved for structural reasons^45^. Since the collagen sequences are distinct between these species, it is likely that the other proteins are as well. Recreating the sequences for these proteins was, however, beyond the scope of this study, since sheep could be distinguished from the wild species based on the differences seen in type 1 collagen.

**Figure 5:**
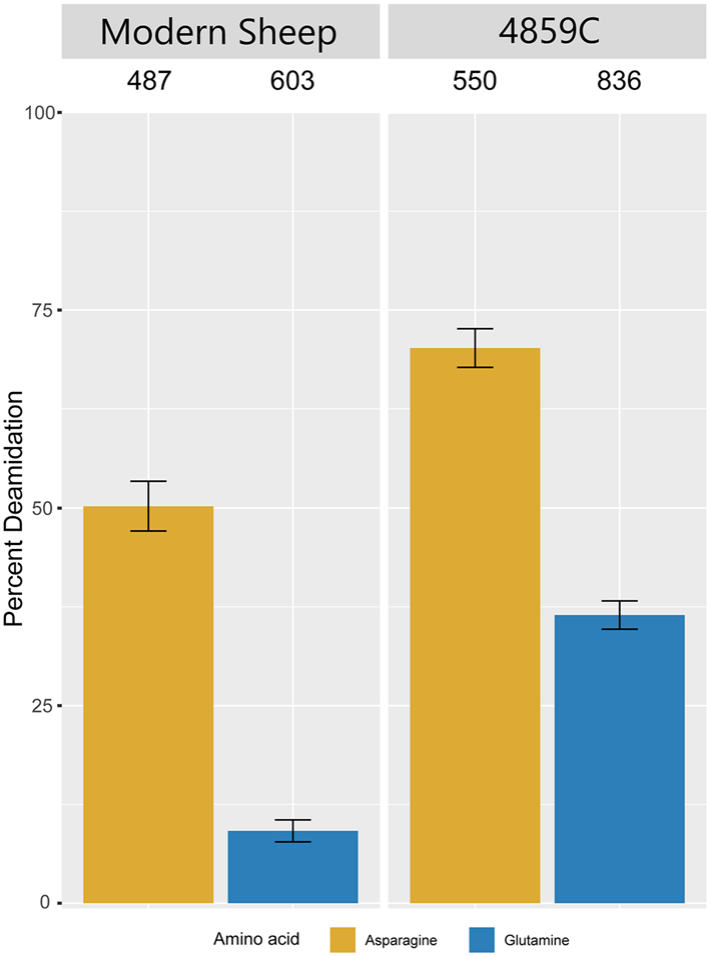
Deamidation levels of modern sheep sample G and Spoegrivier sheep P4859C: Overall percentage of deamidation for asparagine (N) and glutamine (Q) residues for tryptic peptides from archaeological sample P4859C compared with modern sheep bone G, excluding contaminants. Error bars represent standard deviations and the numbers above each bar represent the numbers of peptides on which the calculations are based.

For additional validation, the deamidation levels of the peptides recovered by LC–MS/MS for P4859C and sample G (modern sheep) were calculated (Fig. 5). Asparagine and glutamine residues naturally deamidate over time into aspartic and glutamic acids, respectively. While the rates of these reactions depend on many factors (such as temperature, humidity, and pH^46–48^), higher levels of deamidation are generally associated with older proteins, and hence can help indicate whether the proteins recovered are authentically archaeological, or derive from modern contamination. P4859C shows higher rates of deamidation than the modern sheep sample G, especially for the slower (up to 10X^49^) glutamine reaction, indicating that we recovered and analysed authentically ancient proteins.

## Discussion

ZooMS is an important method to develop in contexts where there is poor preservation of organic remains, as proteins such as collagen can survive longer than DNA in archaeological sites^35,37,50^. Our results provide a framework for using ZooMS to distinguish sheep from the wild species springbok, grey rhebok, and grey duiker. We apply this to remains from archaeological sites in southern Africa with good bone collagen preservation. Using proteomics, our study confirmed that the earliest specimen identified as a domesticate on the basis of its morphology, a first phalanx directly dated to 2105 ± 65 BP, is indeed a sheep. This specimen has now been identified by three independent methods (morphology, ZooMS, LC–MS/MS). We can therefore have confidence that domesticated sheep were indeed introduced into South Africa by ca. 2000 BP.

Recent biomolecular studies at other archaeological sites in this part of the world have shown that some putative early domesticates are actually wild species^24^; see also^16–18^. The winter rainfall climate of south-western Africa precluded the spread of African grain crops (principally sorghum and millets) into the region, so that agriculture was practised only after European colonisation in the seventeenth century CE. The introduction of domesticated animals ca. 2000 years ago therefore marked the transition from a hunter-gatherer world to food production. The timing and mechanism of the arrival of herding is thus a key issue in archaeology, linguistics, population genetics and related fields^14,51–54^.

We successfully identified a ZooMS marker peak at *m/z* 1532 (GEPGP**A**GAVGPAGAVGPR, COL1A2 position 889–906) that was present in all three wild bovids (springbok, grey rhebok, and grey duiker) but not in sheep samples. This marker was validated by LC–MS/MS protein sequencing. ZooMS relies on whole peptide mass (MS1 data), whereas LC–MS/MS fragments this mass into the constituent amino acids (MS2) and determines their order. This allows for unambiguous distinction between peptide masses. We were therefore able to confirm the sequence differences between sheep and the wild bovids, allowing for distinction between the groups. Additionally, through validation of this candidate marker we also constructed COL1A1 and COL1A2 protein sequences for springbok. Thus, this ZooMS marker provides a complementary method to osteological identification.

The consensus springbok collagen sequences that we present differ from those shown in supplementary Fig. S1 of Le Meillour et al. (2020)^24^. That figure shows the GEPGP**V**GAVGPAGAVGPR (corresponding caprine ortholog of the *m/z* 1532 marker) as present in springbok alpha 2 chain of collagen type 1. If correct, this would invalidate our study. To address this issue we re-analysed the LC–MS/MS files provided by Le Meillour et al. (2020). The outcome of this analysis (Supplementary Fig. S3) shows that in fact the GEPGP**V**GAVGPAGAVGPR peptide is *not* present whilst the GEPGP**A**GAVGPAGAVGPR peptide (corresponding to wild bovids) *is* present in the raw data reported by Le Meillour et al. (2020) for their springbok reference material (SPR 89 and SPR 1670), matching our own findings. Note that we are not challenging the species identifications proposed by Le Meillour et al. (2020), which are based on different markers. We merely note an inconsistency between their LC–MS/MS files and their Fig. S1. This demonstrates the need to check every residue in a proposed sequence with at least two different peptide fragments, preferably using more than one software package, as we have done here.

We have also shown that it is possible to identify the ZooMS marker at *m/z* 1532 and others using minimally destructive sampling methods (Supplementary Table S1). Given that many archaeological bones are fragmentary, and specimens of particular interest may have been subjected to other types of destructive analyses, minimally destructive sampling is often desirable. All minimally destructive sampling techniques used in this study produced identifications. Because we tested only six archaeological specimens using these methods, we do not have enough data for a thorough comparison; however, our results show aluminum film gave the most identifications. Further testing is necessary to determine which minimally destructive sampling method is optimal. As PVC eraser is the cheapest and most available option it is likely that it will become the most popular in future studies. Importantly, the bone fragments in our study were not polished or worked, and therefore the collagen probably bound to the PVC eraser or polishing film better than that from highly worked, polished bone surfaces such as those of bone points, which when tested previously with these methods, have had low rates of success^33^. Based on our data, we suggest that continued investigation of minimally destructive sampling techniques is merited; they may in future supplant destructive sampling as the most common strategy. Our results also confirm that it is possible to use archived collagen extracts for ZooMS. Collagen extracted in 1992–3 for radiocarbon dating yielded ZooMS spectra comparable to freshly extracted collagen, and was suitable for LC–MS/MS protein sequencing. We were also able to detect other proteins (such as alpha-2-HS-glycoprotein and osteocalcin), further expanding the utility of archived collagen. Archived collagen may be available from important directly dated samples for which there is no more bone available, or on which curators are unwilling to allow further destructive analysis.

As applications of ZooMS expand in different parts of the world with different fauna, ongoing developmental work is required to build and validate reference peptide fingerprint libraries to investigate whether it is possible to distinguish species of interest using the peptide fingerprinting method. In this paper, we have taken a step towards this goal, focussing on domesticated sheep and co-occurring wild species in south-western Africa. Future studies will benefit from this diagnostic ZooMS marker to distinguish between sheep and wild African bovids of similar size. This type of work is essential to develop a reliable picture of the spread of herding in southern Africa and elsewhere.

## Materials and methods

For a complete description of all methodology see supplemental methods.

### Samples

For this investigation, we examined reference bone samples from South African sheep, goat, and three wild bovid species to obtain peptide fingerprints for comparison with archaeological samples from four sites (Fig. 1). We analysed both small fragments of bone (< 100 mg), the removal of which slightly alters the overall morphology of the specimen, and ‘minimally destructive samples’ by which we mean eraser/film rubbings, as described below. We define ‘minimally destructive’ as sampling that does not affect the overall morphology of the specimen(s). A list of samples is provided in Table 1.

### Protein extraction

Protein extraction was performed on samples derived from destructive sampling according to the protocol described in Buckley et. al. (2009)^36^, with minor modification.

### ZooMS

ZooMS analysis was performed on all destructive and minimally destructive samples following the protocols described in Buckley et al. (2009)^36^; Fiddyment et al. (2015)^38^; Kirby, Manick and Newman (2019)^40^. Where possible 20 µg of protein and a trypsin:protein ratio (µg) of 1:50 was used. Instrument and settings were typical for archaeological samples and the same as described in Jensen et al. (2020)^55^.

### LC–MS/MS acquisition

The reference samples A (springbok), D (grey rhebok), G (Namaqua Afrikaner sheep), and M (grey duiker), as well as collagen from the earliest dated archaeological sample (P4859C) from Spoegrivier were further analysed by LC–MS/MS following methods previously published for palaeoproteomic samples^55,56^. Tryptic and elastase digestions for each sample were combined into a single mass spectrometry run.

### LC–MS/MS data analysis

To validate the presence of the suspected ZooMS markers, the Thermo RAW files generated were then searched using the software MaxQuant (v.1.6.3.4)^57^. The database was prepared using previously published type 1 collagen sequences from several species including *Ovis aries, Capra hircus*, *Bos taurus, Mus musculus,* and *Homo sapiens*. For MaxQuant settings see supplemental methods. Proteins were considered confidently identified if at least two razor + unique peptides covering distinct areas of the sequence were recovered. MS/MS spectra were assessed manually for confident identification. Deamidation was assessed using publicly available code^56^, with the contaminant proteins filtered out. In addition, the archaeological sample P4859C was searched in MaxQuant against the sheep proteome database from Uniprot (downloaded 20/3/20) in order to identify other proteins besides COL1.

### Construction of springbok collagen sequence

The raw file created from modern springbok bone (A) was also searched with PEAKS (v.7)^58^ against a database comprising COL1 sequences of the following species: *Ovis aries, Capra hircus, Bos taurus, Sus scrofa, Mus musculus,* and *Homo sapiens.* The searches (de novo, PEAKS DB, PEAKS PTM, and SPIDER) were performed with peptide mass tolerance + /− 10 ppm and fragment mass tolerance + /− 0.05 Da. SAPs were then authenticated by searching the sample of modern springbok bone (A) with MaxQuant (v 1.6.3.4) using the same database as described above, but with the added springbok predicted sequences. These trypsin and elastase searches were repeated and the springbok sequence(s) updated until a single predicted springbok sequence remained that incorporated all SAPs confidently detected.

The confidently matched spectra were then aligned using MAFFT’s online service^59^ (v.7) and a consensus sequence was then made from these peptides. An amino acid residue was only considered correctly assigned if it could be confidently identified in at least two overlapping peptides. All residues that did not reach this standard are marked as X in the final sequence. The final sequence is provided in the supplementary results.

## Supplementary Information

Supplementary Information 1.

Supplementary Information 2.

## Data Availability

The mass spectrometry proteomics data (including the averaged MALDI-TOF TXT files) have been deposited to the ProteomeXchange Consortium via the PRIDE^60^ partner repository with the dataset identifier PXD021949.
