# Supplementary material for: Palaeoproteomics confirm earliest domesticated sheep in southern Africa ca. 2000 BP
Source: Sci Rep. 2021 Mar 23;11:6631. doi: 10.1038/s41598-021-85756-8 (PMC7988125; doi:10.1038/s41598-021-85756-8)
Supplement: Supplementary file 1 — Supplementary Information 1. [file 41598_2021_85756_MOESM1_ESM.pdf]

Palaeoproteomics confirm earliest domesticated sheep in southern Africa ca. 2000 BP

Authors

Ashley N. Coutu[1,2,6]*, Alberto J. Taurozzi[3]*†, Meaghan Mackie[3,4], Theis Zetner Trolle Jensen[3], Matthew J. Collins[3,5] & Judith Sealy[6]†

***** these authors contributed equally to the work
† corresponding authors

Supplementary Information

1. Supplemental Methods

1.1 Protein Extraction

Archaeological and modern bone fragments were treated initially with 0.6 M hydrochloric acid (HCl) for several days at 4 °C to demineralise the sample before solubilisation of the protein fraction. After demineralisation, the supernatant was removed, 150 µl of 50 mM ammonium bicarbonate (Ambic) was added, vortexed, and then centrifuged for 1 minute. This process was repeated 2 times. For the final rinse, the pH of the supernatant was checked to verify that the HCl had been successfully removed. Archaeological and modern bone fragments were then solubilised by incubation with 100 µl of 50 mM Ambic at 65 °C for 1 hour. After solubilisation the extracted protein concentration was determined using BCA (bicinchoninic acid) assay according to the manufacturer's instructions. Briefly, a 5 point standard series was made by serially diluting bovine serum albumin (BSA) covering a concentration range of 62.5 - 1000 µg/ml. Each sample was measured in duplicate with 10 µl of sample incubated with 200 µl of BCA 'working reagent' at 37 °C before absorbance at 560 nm was measured using a plate reader. Where possible a volume of sample extraction containing 20 µg of protein was transferred into a new protein Eppendorf LoBind tube and the volume adjusted to a minimum volume of 50 µl with 50 mM Ambic, along with 1 µl of 0.4 µg/µl of sequencing grade trypsin (Promega). Trypsin digestion was performed at 37 °C overnight with mild shaking (500 rpm). After digestion, the samples were centrifuged at 10,000 g for 1 minute and then acidified to < pH 2 using 5% (v/v) trifluoroacetic acid (TFA). Peptide clean-up was performed using C18 reverse phase resin ZipTips according to the manufacturer's instructions. Peptides were eluted using 50 µl of 50% acetonitrile (ACN) 0.1% TFA solution.

A subset of the archaeological and modern bone fragments were also sampled using two minimally destructive techniques: PVC eraser [1,2] and polishing films [3]. The PVC eraser method involves rubbing the bone with a small fragment of eraser, and through triboelectric friction, the collagen is transferred from the bone to the eraser. The resultant eraser crumbs are collected into a LoBind Eppendorf tube. The polishing film method involves rubbing the bone with a

small piece of polishing film with a gritted surface. The films come in a range of different grit sizes and materials (1–30 µm particles); here we used 15 µm alumina film and 3 µm diamond film, held in tweezers. Friction of the film against the bone surface creates abrasion and microscopic bone particles are transferred to the film, which is then placed whole into an Eppendorf tube. For both PVC eraser and polishing film methods, 75 µL of Ambic was added directly to the eraser rubbings/film pieces in the tube with 1 µl of 0.4 µg/µl of sequencing grade trypsin (Promega). Trypsin digestion was performed at 37 °C for four hours. After digestion, the samples were centrifuged at 10,000 g for 1 minute and then acidified to < pH 2 using 5% (v/v) TFA. Peptide clean-up was performed using C18 reverse phase resin ZipTips according to the manufacturer's instructions. Peptides were eluted using 50 µl of 50% ACN 0.1% TFA solution.

1.2 Matrix-assisted Laser Desorption/Ionization Time-of-Flight Mass Spectrometry data acquisition and analysis

Peptide eluates from all extraction methods (solution, PVC eraser, polishing film) were co crystallised with α-cyano-4-hydroxycinnamic acid (Sigma Aldrich) matrix solution (50% ACN /0.1% TFA (vol/vol) at a ratio of 1:1 (1µL : 1µL). Mass spectrometry was performed using a Bruker Ultraflex III (Bruker Daltonics) matrix-assisted laser desorption/ionization time of flight mass spectrometer (MALDI-TOF-MS) run in reflector mode with laser acquisition set to 1200 and acquired over an *m/z* range of 800–3200. The generated spectral output was converted to TXT and was analysed using the open-source software mMass v.5.5.0 [4]. The triplicate raw files were merged, and then peak picked with a S/N threshold of 4. MALDI-TOF-MS was performed at the Centre for Excellence in Proteomics at the University of York, United Kingdom.

1.3 Liquid Chromatography Tandem Mass Spectrometry data acquisition and analysis *1.3.1 LC-MS/MS Acquisition*

The reference samples A (springbok), D (grey rhebok), G (Namaqua Afrikaner sheep), and M (grey duiker), as well as collagen from the earliest dated archaeological sample (P4859C) from Spoegrivier were further analysed by LC-MS/MS. Samples were processed as for ZooMS but an additional parallel digestion was performed with elastase instead of trypsin. The tryptic and elastase ZipTip eluates were combined before being evaporated to dryness using a vacuum concentrator (Eppendorf, Hamburg, Germany), and transferred to the Novo Nordisk Foundation Center for Protein Research, the University of Copenhagen for LC-MS/MS analysis on a EASY nLC 1200 (Proxeon, Odense, Denmark) coupled to a Q-Exactive HF-X (Thermo Scientific, Bremen, Germany). The volume required for approximately 2 µg of protein per sample was placed in separate wells on a new 96-well plate and topped up to 30 µL using 40% ACN and 0.1% formic acid (FA) They were then vacuum centrifuged and resuspended with 10 µL of 0.1% TFA, 5% ACN, and 5 µL of sample analysed by LC-MS/MS on a 77 min gradient. The LC MS/MS parameters were the same as previously used for palaeoproteomic samples [5,6], in short: MS1: 120 k resolution, maximum injection time (IT) 25 ms, scan target 3E6. MS2: 60 k

resolution, top 10 mode, maximum IT 118 ms, minimum scan target 3E3, normalised collision energy of 28, dynamic exclusion 20 s, and isolation window of 1.2 *m/z*.

1.3.2 LC-MS/MS Data Analysis

To validate the presence of the suspected ZooMS markers, the Thermo RAW files generated were then searched using the software MaxQuant (v.1.6.3.4) [7]. The database was prepared using previously published type 1 collagen sequences from several species including *Ovis aries*, *Capra hircus*, *Bos taurus*, *Mus musculus*, and *Homo sapiens*. MaxQuant settings were as follows: Digestion mode was set to semi-specific for trypsin, to account for possible additional hydrolytic cleavages occurring during diagenesis. Variable modifications were: oxidation (M), acetyl (Protein N-term), deamidation (NQ), Gln→pyro-Glu, Glu→pyro-Glu, and hydroxyproline. Carbamidomethyl (C) was set as a fixed modification. The remaining settings were set to the program defaults, apart from Min. score for unmodified and modified peptides searches, which were both set to 60. Proteins were considered confidently identified if at least two razor+unique peptides covering distinct areas of the sequence were recovered. MS/MS spectra were assessed manually for confident identification. In addition, the samples were searched against the MaxQuant contaminant database that identifies proteins which may be present due to sample handling and laboratory analysis. Any protein not considered authentic (i.e. keratins from skin, the laboratory standard BSA) was not included in further analysis. Deamidation was assessed using publicly available code [6], with the contaminant proteins filtered out.

In addition, the archaeological sample Spoegrivier P4859C was searched in MaxQuant against the sheep proteome database from Uniprot (downloaded 20/3/20) in order to identify other proteins besides COL1. This was done once with the same MaxQuant settings as above and then again for semi specific digestion with elastase instead of trypsin, and the results combined (Table. S3 and S4). Species specific peptides for the proteins recovered were identified as specific based on searches using the NCBI BLASTp tool (https://blast.ncbi.nlm.nih.gov/Blast.cgi) against all publicly available protein sequences.

1.3.3 Construction of springbok collagen sequence

Springbok is the species most likely to be confused with sheep in this context, especially at Spoegrivier. Therefore, the collagen 1 (COL1) sequence for springbok was derived from the LC MS/MS springbok sample. The raw file created from modern springbok bone (A) was also searched with PEAKS (v.7)[8] against a database comprising COL1 sequences (COL1A1 and COL1A2 were combined with a single K residue separating them) of the following species: *Ovis aries*, *Capra hircus*, *Bos taurus*, *Sus scrofa*, *Mus musculus*, and *Homo sapiens.* The searches (de novo, PEAKS DB, PEAKS PTM, and SPIDER) were performed with peptide mass tolerance +/- 10 ppm and fragment mass tolerance +/- 0.05 Da. No enzyme was specified. Searches allowed the variable modifications for deamidation (NQ), oxidation (M), Gln ->pyro-Glu, Glu ->pyro Glu, and hydroxyproline. Any single amino acid polymorphism (SAP) with

convincing evidence
(more than half of the residues located at that position with well annotated spectra) detected through the searches were compiled into a new COL1 sequence for springbok. In cases where it could be ambiguous, two versions of the sequence were made.

These SAPs were then authenticated by searching the sample of modern springbok bone (A) with MaxQuant (v 1.6.3.4) using the same database as before, but with the added springbok predicted sequences. Digestion mode was set to semi-specific for trypsin. Variable modifications were: oxidation (M), deamidation (NQ), Gln →pyro-Glu, Glu →pyro-Glu, and hydroxyproline. Carbamidomethyl (C) was set as a fixed modification. The remaining settings were set to the program defaults, apart from minimum score for unmodified and modified peptides searches, which were both set to 60 to limit the amount of poor quality spectra included in the analysis. This search was also repeated for the semi-specific search of elastase, and those results combined with that of the trypsin search. These trypsin and elastase searches were repeated and the springbok sequence(s) updated according to well annotated matches from the other sequences in the database, until one predicted springbok sequence was left that incorporated all SAPs confidently detected.

Each peptide assigned to springbok COL1 was then manually examined for well annotated spectra, with the consideration that for most peptides every third residue would be a glycine (G), as that is the structure of the vast majority of the collagen sequence [9]. If part of the sequence was in doubt due to poor annotation and/or missing ions, these residues would be changed to an X. This included the consideration of post-translational modifications (PTMs) that could affect the interpretation of the sequence. This was mostly focused on the inspection of deamidated residues (where aspartic acid could be deamidated asparagine and glutamic acid deamidated glutamine). If all spectra of a peptide contained the deamidated form of a residue it was marked with an X. Additionally, the mass of a hydroxylated proline (common in collagen) beside an alanine has the same mass as an unmodified proline and a serine. In this case the true sequence can only be known if these residues are detected in separate ion fragments. If this was not possible, the two residues were replaced with Xs.

The confidently matched spectra (including those with X residues but otherwise well annotated), were then aligned using MAFFT's online service [10] (v.7). A consensus sequence was then made from these peptides, with each amino acid residue considered correctly assigned only if it could be confidently identified in at least two overlapping peptides. All residues that did not reach this standard were marked with an X in the final sequence. In addition, since leucine and isoleucine cannot be distinguished with this protocol (being the same mass in a different configuration), all isoleucines (I) from the original sequence have been presented as leucines (L) for consistency[11].
The peptides used for reconstruction and the resulting predicted and final springbok sequences (separated into COL1A1 and COL1A2) are provided below (and Table S2).

Final searches (both semi specific for trypsin and elastase) of springbok bone A and the other modern reference bones (D, G, M) as well as the archaeological sample Spoegrivier 4859C, were performed with the final springbok predicted sequence database for comparison between

these samples, and for additional validation of the presence of the suspected ZooMS marker of interest.

2. ZooMS spectra images from 4 reference samples and Spoegrivier P4859C.

Figure S1: *Collagen peptide mass fingerprints for modern reference samples A (a.), D (b.), G (c.), M (d.), and archaeological sample Spoegrivier P4859C (e.)*

a.) Springbok (A)

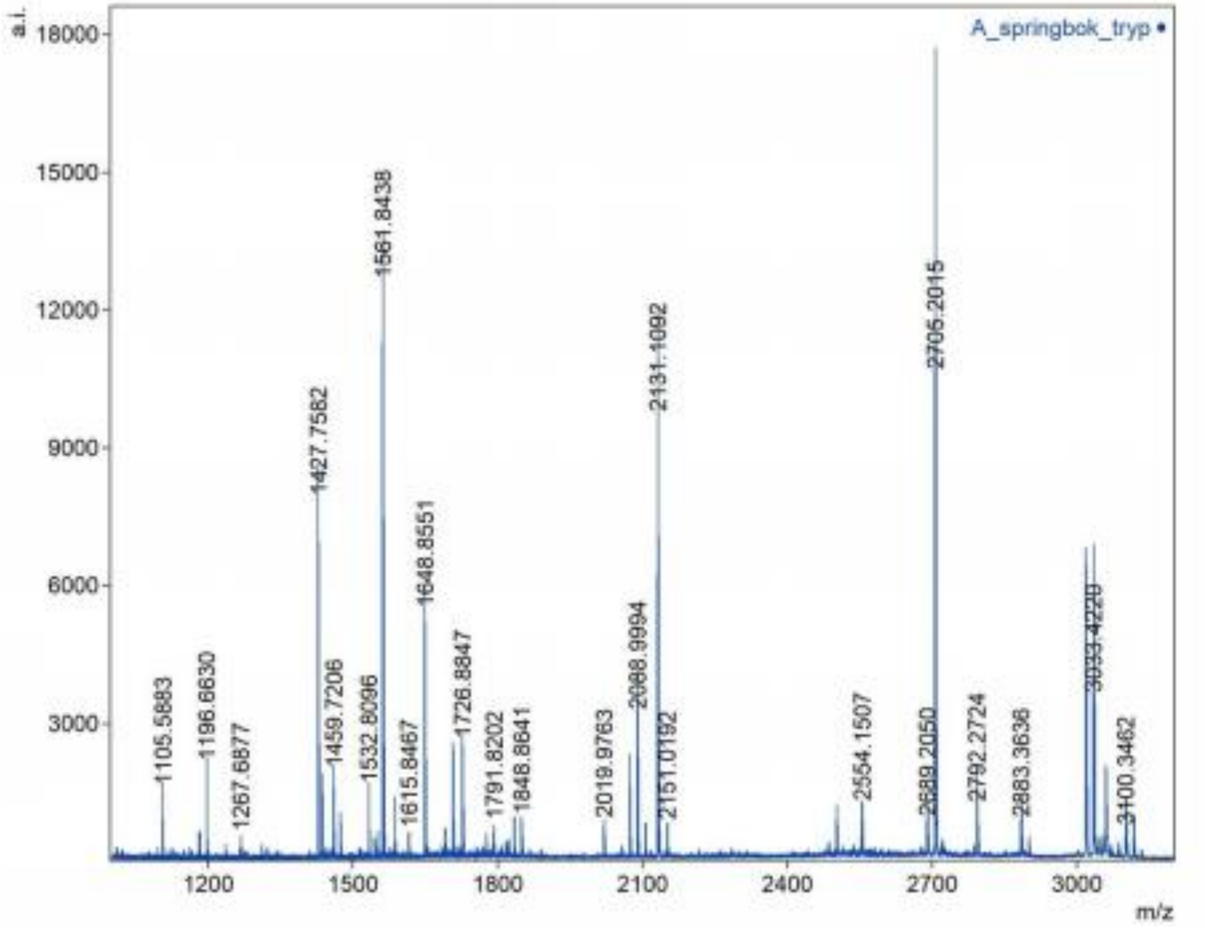

b.) Grey rhebok (D)

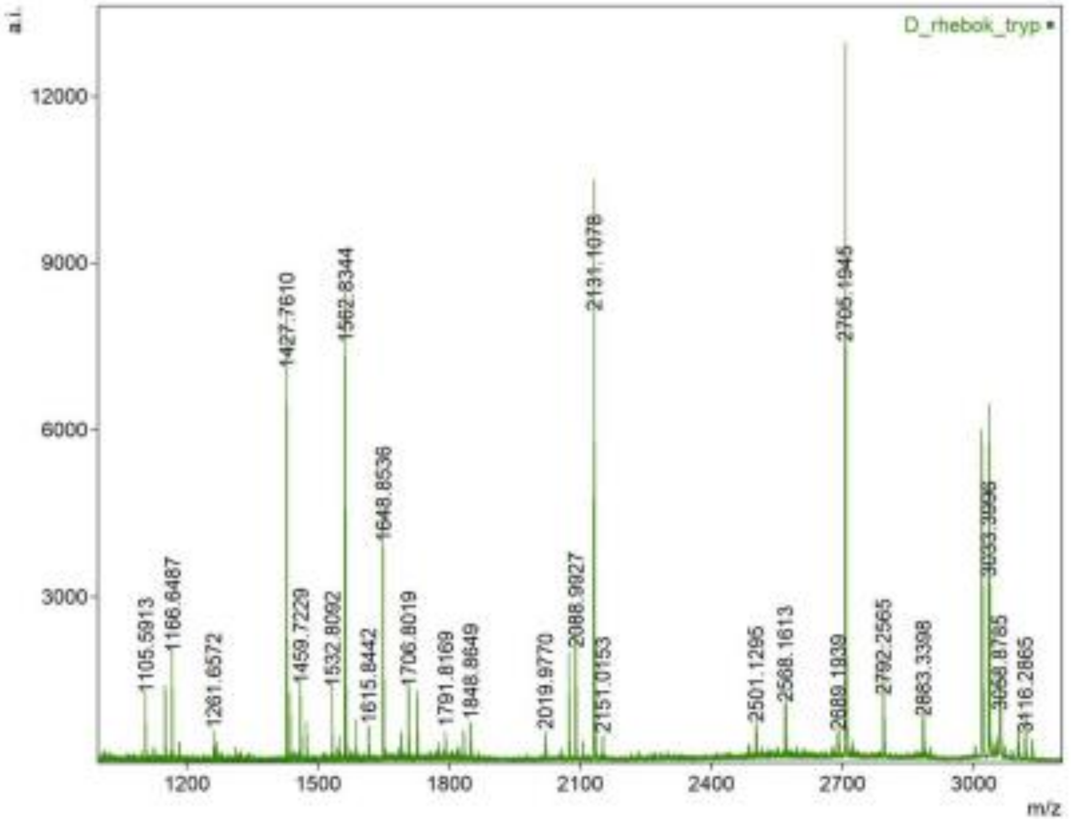

c.) Namaqua Afrikaner sheep (G)

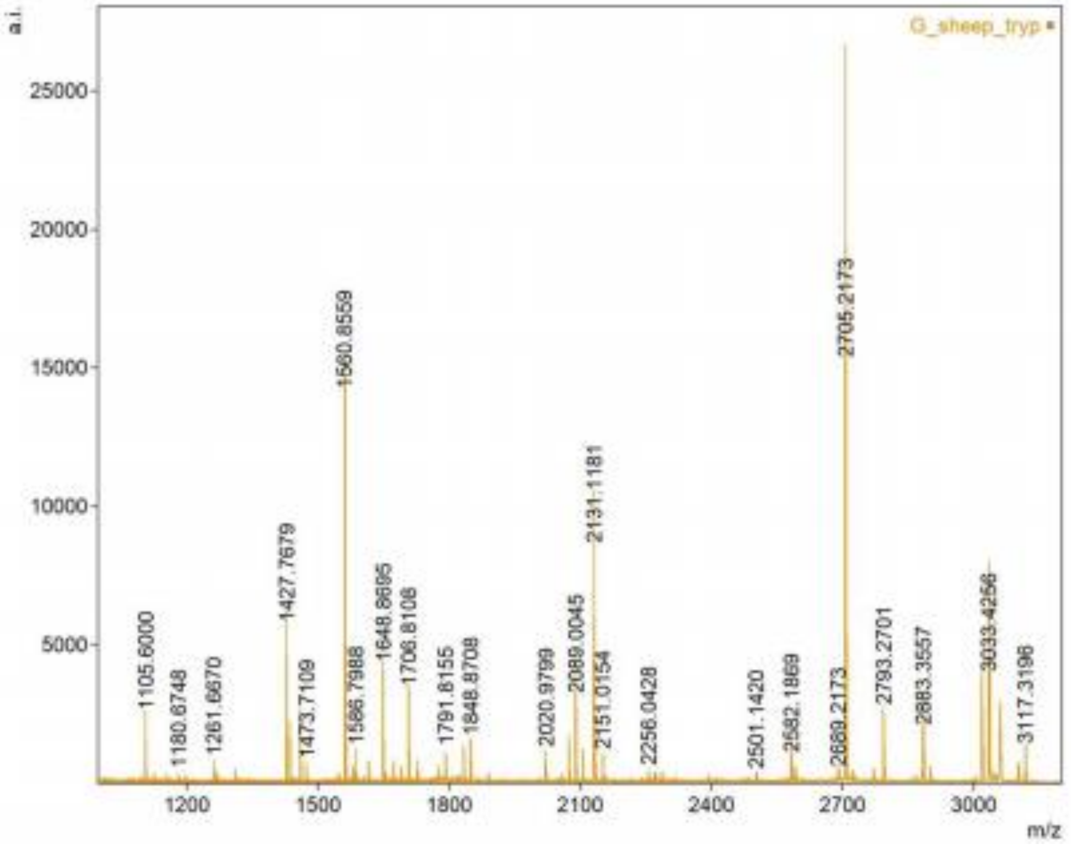

d.) Grey duiker (M)

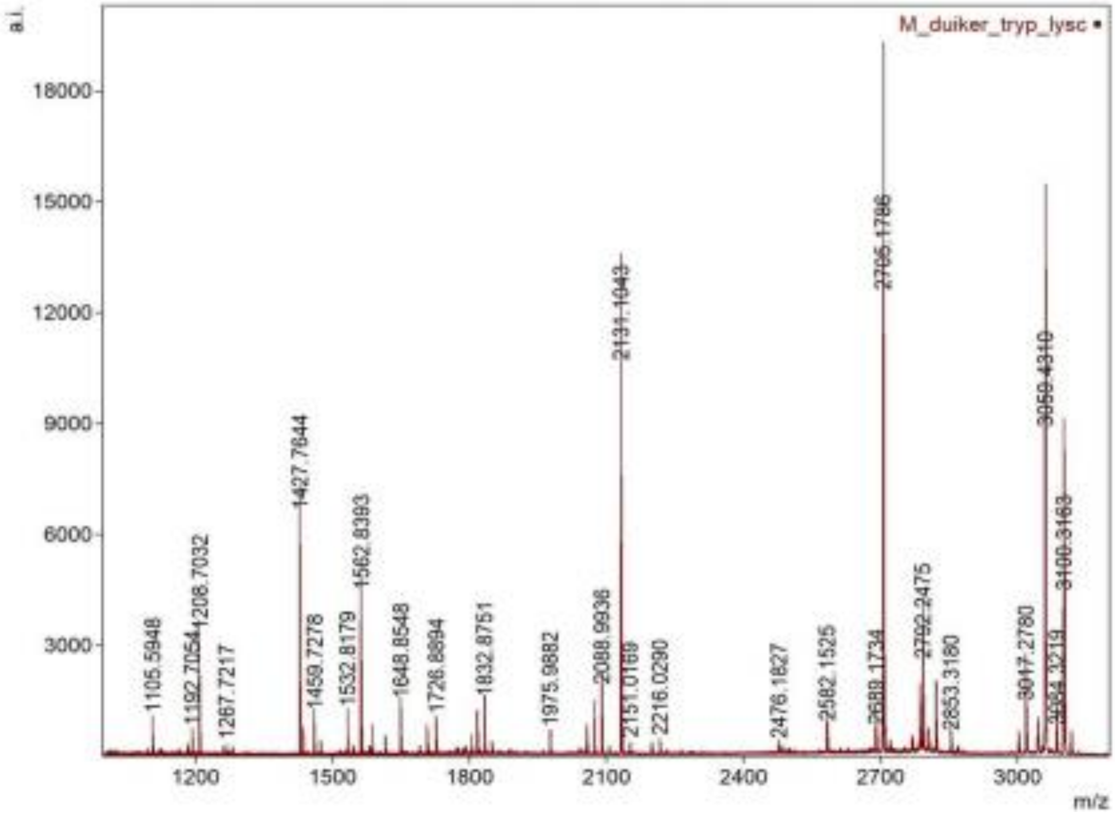

e.) Spoegrivier P4859C (sheep)

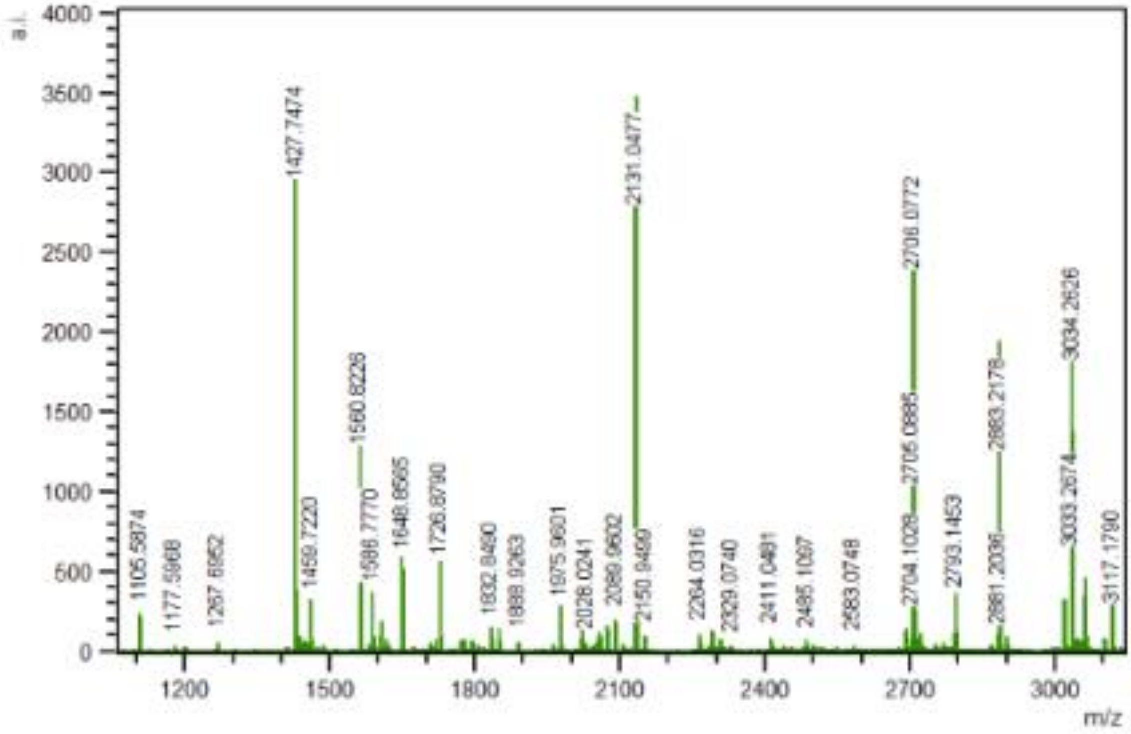

3. ZooMS markers

Table S1: ZooMS *m/z* markers for each method tested: *It is necessary to screen ZooMS spectra produced from minimally destructive sampling techniques (eraser and film methods) for common contaminants such as human keratin (from skin, nails or hair) deriving from handling during or post excavation, and the plastic residue of the eraser and films themselves. These contaminant peaks have been removed from the analysed spectra, following[123]. As reported in McGrath et al. 2019, keratin peaks are often a higher intensity than sample peaks. Letter of the corresponding peptide marker is listed in the header row for each m/z value using the lettering system of naming peptides used in Buckley et al. 2009, with the newly proposed nomenclature of Brown et al. 2020 in brackets: P1= (COL 1 α1 508-519); A1= (COL 1 α2 978-990); A2= (COL 1 α2 978-990) B= (COL 1 α2 484-498); C= (COL 1 α2 502-519); P2= (COL 1 α2 292-309); D= (COL 1 α2 793-816); E= (COL 1 α2 454-483); F1= (COL 1 α1 586-618); F2= (COL 1 α1 586-618); G1= (COL 1 α2 757-789); G2= (COL 1 α2 757-789). These peptide markers have only been verified against LC-MS/MS sequence data for springbok (this study), sheep and goat[13]. The m/z markers for grey rhebok and grey duiker are italicised because the peptide markers are predicted from reference spectra generated by this study, but no LC-MS/MS sequence data have verified the peptide markers corresponding to the m/z values for these two species. * in the Method column means the spectra were poor. ^ in the Method column means there were keratin peaks present in the spectra.*

Sample ID	Method	ZooMS ID	P1 (α1 508)	A1 (α2 978)	A2 (α2 978) A1 +16	B (α2 484)	(α2 889)	C (α2 502)	P2 (α2 292)	D (α2 793)	E (α2 454)	F1 (α1 586)	F2 (α1 586) F1 +16	G1 (α2 757)	G2 (α2 757) G1 +16
A	Eraser *^	springbok	1105	1180		1427	1532								
	Aluminum Film (15µm)	springbok	1105	1180		1427	1532	1550	1648	2131		2883		3017	
	Diamond Film (3µm)	springbok	1105	1180	1196	1427	1532	1550	1648	2131		2883			
	Bone fragments	springbok	1105	1180	1196	1427	1532	1550	1648	2131	2792	2883	2899	3017	3033
B	Eraser ^	springbok	1105	1180	1196	1427	1532	1550	1648	2131		2883		3017	
	Aluminum Film (15µm)	springbok	1105	1180	1196	1427	1532	1550	1648	2131		2883		3017	3033
	Diamond Film (3µm)	springbok	1105	1180	1196	1427	1532	1550	1648	2131		2883		3017	

	Bone fragments	springbok	1105	1180	1196	1427	1532	1550	1648	2131	2792	2883		3017	3033
C	Eraser ^	springbok	1105	1180	1196	1427	1532	1550	1648	2131					
	Aluminum Film (15μm)	springbok	1105	1180	1196	1427	1532	1550	1648	2131		2883	2899	3017	3033
	Diamond Film (3μm)	springbok	1105	1180	1196	1427	1532	1550	1648	2131		2883	2899	3017	3033
	Bone fragments	springbok	1105		1196	1427	1532	1550	1648	2131	2792	2883	2899	3017	3033
D	Eraser *	grey rhebok													
	Aluminum Film (15μm) *	grey rhebok													
	Diamond Film (3μm)	grey rhebok	*1105*	*1150*	*1166*	*1427*	*1532*	*1550*	*1648*	*2131*		*2883*	*2899*		
	Bone fragments	grey rhebok	*1105*	*1150*	*1166*	*1427*	*1532*	*1550*	*1648*	*2131*	*2792*	*2883*		*3017*	*3033*
M	Bone fragments	grey duiker	*1105*	*1192*	*1208*	*1427*		*1580*	*1648*	*2131*	*2792*	*2853*		*3043*	*3059*
N	Bone fragments	grey duiker	*1105*	*1192*	*1208*	*1427*			*1648*	*2131*	*2792*	*2853*		*3043*	*3059*
F	Eraser	sheep *													
	Aluminum Film (15μm)	sheep	1105		1196	1427		1580	1648	2131	2792	2883	2899	3017	3033
	Diamond Film (3μm)	sheep	1105	1180	1196	1427		1580	1648	2131	2792	2883	2899	3017	3033
	Bone fragments	sheep	1105		1196	1427			1648	2131	2792	2883		3017	3033
G	Bone fragments	sheep	1105	1180	1196	1427		1580	1648	2131	2792	2883	2899	3017	3033

I	Bone fragments	sheep	1105	1180	1196	1427			1648	2131		2883	2899	3017	3033
J	Bone fragments	sheep	1105	1180	1196	1427		1580	1648	2131	2792	2883	2899	3017	3033
K	Bone fragments	goat	1105	1180		1427			1648	2131	2792	2883		3017	3093
L	Bone fragments	goat	1105	1180		1427			1648	2131	2792	2883		3017	3093
P4859	Eraser *	sheep	1105		1196	1427			1648	2131		2883			
	Aluminum Film (15μm)	sheep	1105		1196	1427		1580	1648	2131		2883		3017	3033
	Diamond Film (3μm)	sheep	1105	1180	1196	1427		1580	1648	2131		2883			3033
	Bone fragments	sheep	1105		1196	1427		1580	1648	2131	2792	2883	2899	3017	3033
P4859C	Collagen	sheep	1105		1196	1427		1580	1648	2131	2792	2883	2899	3017	3033
P4863	Eraser * ^	sheep	1105	1180	1196	1427		1580	1648						
	Aluminum Film (15μm) * ^	sheep	1105			1427		1580	1648	2131		2883			
	Diamond Film (3μm)	sheep	1105	1180	1196	1427		1580	1648	2131		2883			
	Bone fragments	sheep	1105	1180	1196	1427		1580	1648	2131	2792	2883	2899	3017	3033
P4864	Eraser	sheep	1105	1180	1196	1427		1580	1648	2131		2883			
	Aluminum Film (15μm)	sheep	1105	1180		1427		1580	1648	2131		2883			

	Diamond Film (3µm)	sheep	1105	1180	1196	1427		1580	1648	2131		2883		3017	3033
	Bone fragments	sheep	1105		1196	1427		1580	1648	2131	2792	2883		3017	3033
P4772	Bone fragments	sheep	1105	1180		1427		1580	1648	2131	2792	2883	2899	3017	3033
P4773	Bone fragments	sheep	1105			1427		1580	1648	2131	2792	2883	2899	3017	3033
P4862	Eraser *														
	Aluminum Film (15µm) *														
	Diamond Film (3µm) *														
	Bone fragments *														
P4861	Eraser	sheep	1105	1180	1196	1427		1580	1648	2131		2883		3017	3033
	Aluminum Film (15µm)	Sheep	1105			1427		1580	1648	2131		2883		3017	3033
	Diamond Film (3µm) * ^														
	Bone fragments	sheep	1105			1427			1648	2131	2792	2883	2899	3017	3033
P4865	Eraser * ^														
	Aluminum Film (15µm)	sheep	1105	1180		1427		1580	1648	2131					
	Diamond Film (3µm) *														
	Bone fragments	sheep	1105	1180	1196	1427		1580	1648	2131	2792	2883	2889	3017	3033

4. *m/z* 1532 Marker Verification

Figure S2: *Wild Bovid (GEPGP***A***GAVGPAGAVGPR) or Caprine (GEPGP***V***GAVGPAGAVGPR) marker verification in modern samples A, D, G, M, and archaeological sample Spoegrivier P4859C.*

a.) Springbok (A)

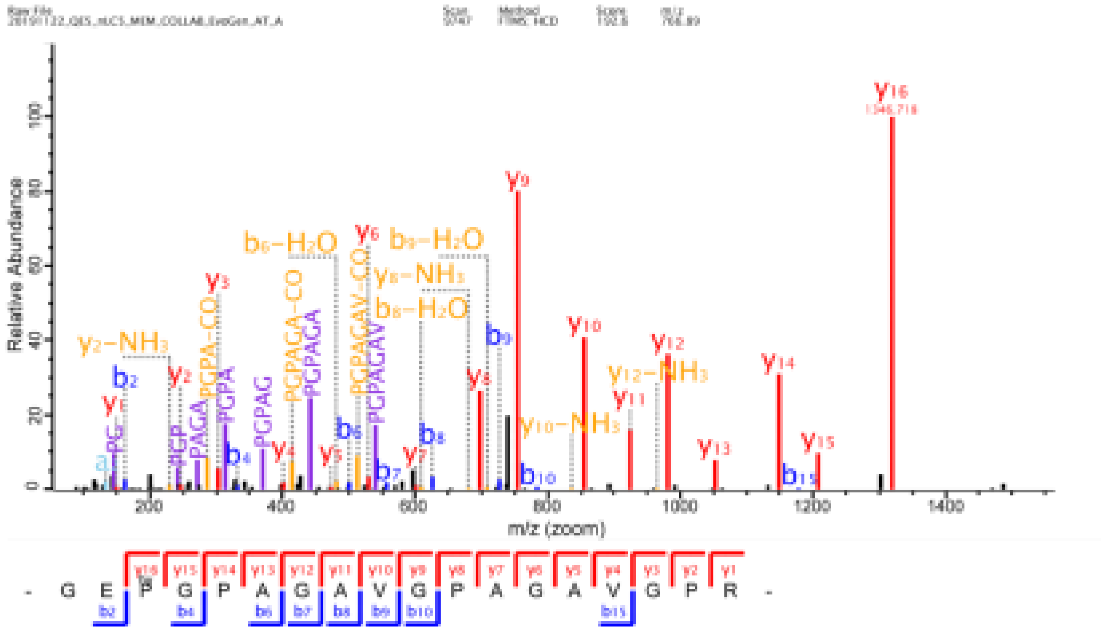

b.) Grey rhebok (D)

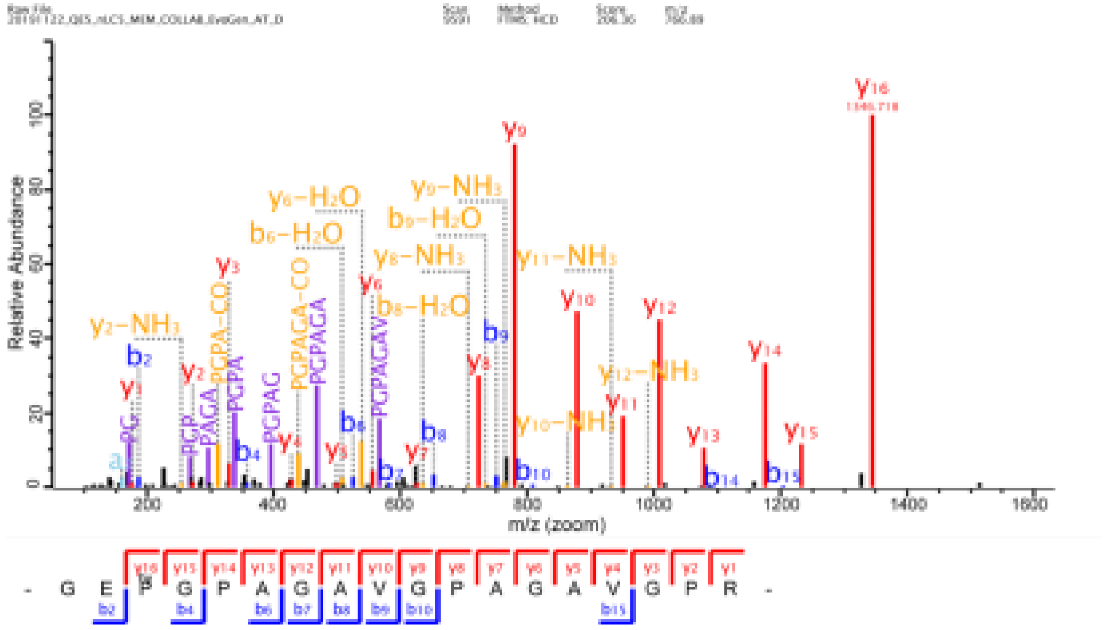

c.) Afrikaner sheep (G)

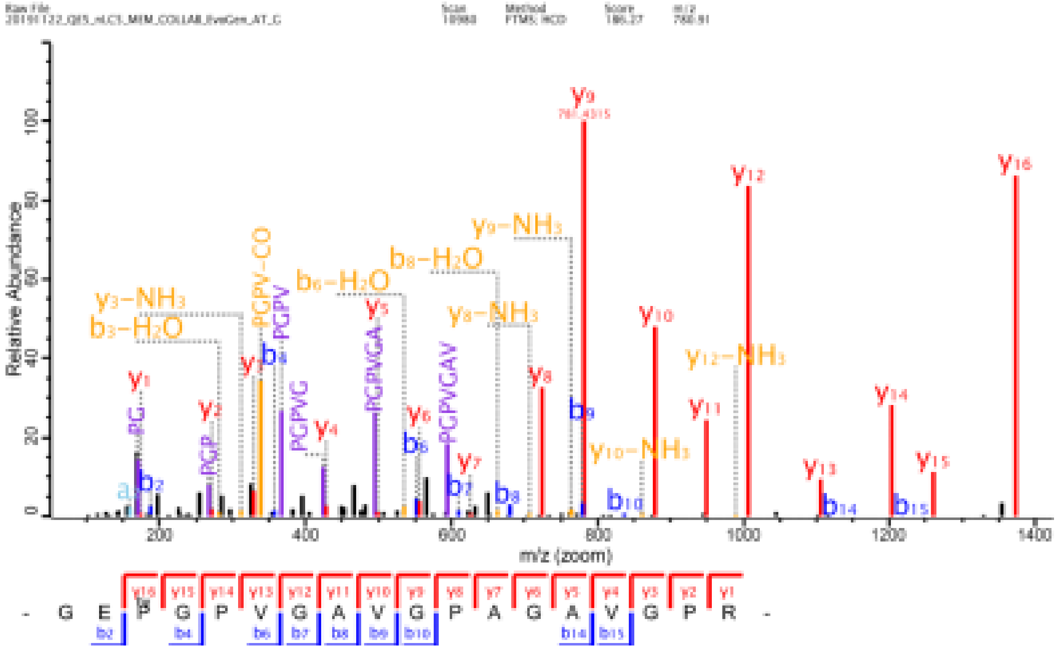

d.) Grey duiker (M)

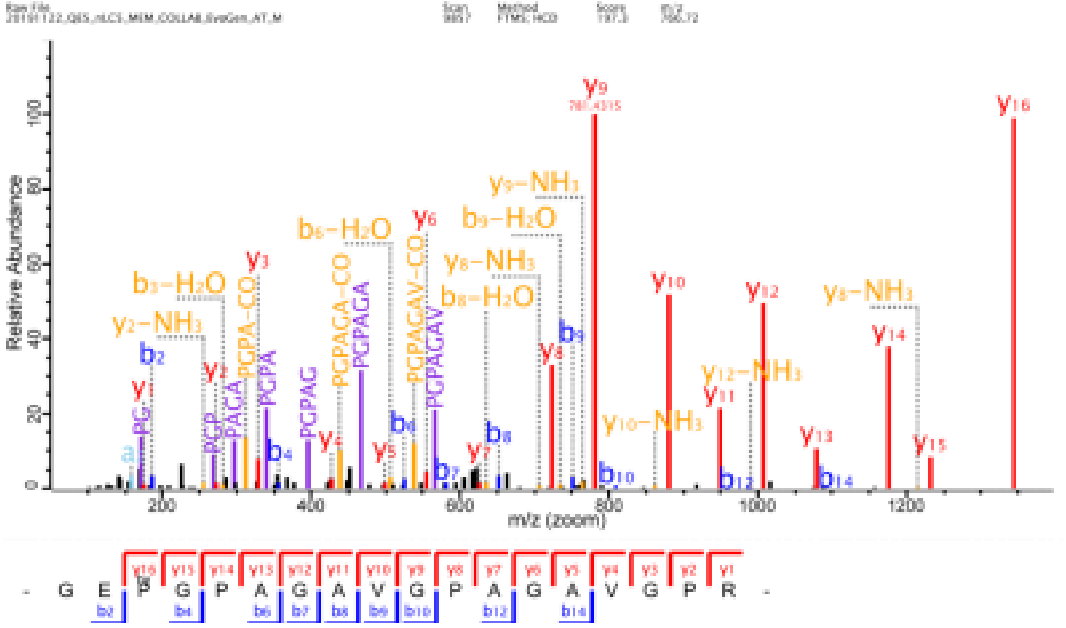

e.) Spoegrivier P4859C (sheep)

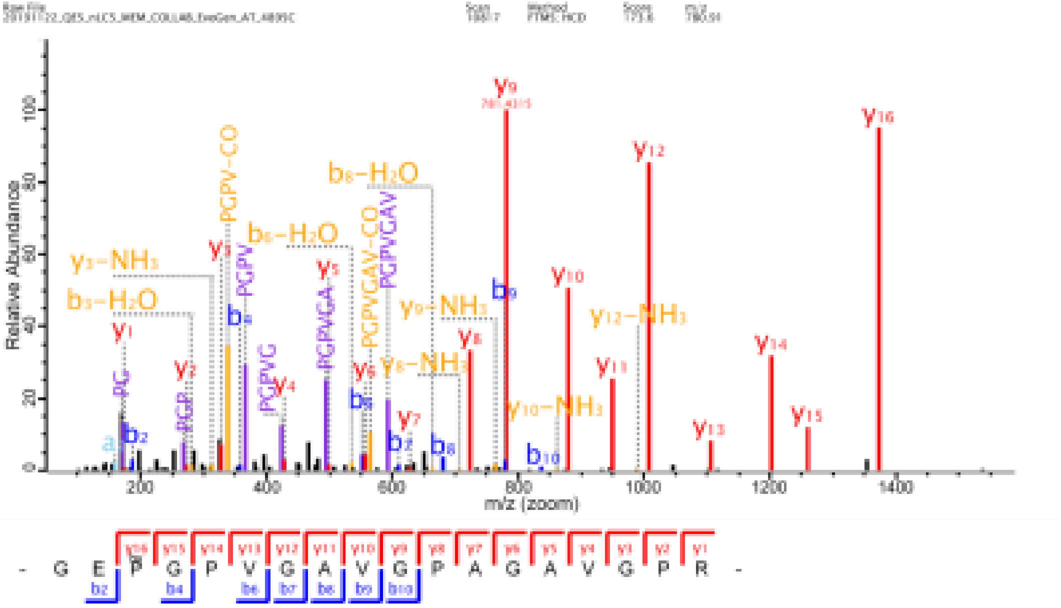

5. Springbok Collagen 1 Sequences

All peptides used from the MaxQuant tryptic and elastase searches of Springbok A are provided in a supplemental Excel spreadsheet (Table S2). Searches are also uploaded to the PRIDE repository with the raw files.

a. ***Coverage of proposed springbok COL1 sequence from PEAKS and MaxQuant analyses***. *Red indicates that amino acid was recovered confidently only once; white with black background indicates an amino acid was not recovered at all or confidently*

>Preliminary_Springbok_A_COL1A1

QLSYGYDEKSTGISVPGPMGPSGPRGLPGPPGAPGPQGFQGPPGEPGEPGASGPMGPRGPPGPPGKNGDDGEAGK
P
GRPGERGPPGPQGARGLPGTAGLPGMKGHRGFSGLDGAKGDAGPAGPKGEPGSPGENGAPGQMGPRGLPGERG
RPGAPGPAGARGNDGATGAAGPPGPTGPAGPPGFPGAVGAKGEAGPQGPRGSEGPQGVRGEPGPPGPAGAAGPA
GNPGADGQPGAKGANGAPGIAGAPGFPGARGPSGPQGPSGPPGPKGNSGEPGAPGSKGDTGAKGEPGPTGIQGPP
GPAGEEGKRGARGEPGPAGLPGPPGERGGPGSRGFPGADGVAGPKGPAGERGAPGPAGPKGSPGEAGRPGEAGL
PGAKGLTGSPGSPGPDGKTGPPGPAGQDGRPGPPGPPGARGQAGVMGFPGPKGAAGEPGKAGERGVPGPPGAVG
PAGKDGEAGAQGPPGPAGPAGERGEQGPAGSPGFQGLPGPAGPPGEAGKPGEQGVPGDLGAPGPSGARGERGFP
GERGVQGPPGPAGPRGANGAPGNDGAKGDAGAPGAPGSQGAPGLQGMPGERGAAGLPGPKGDRGDAGPKGAD
GAPGKDGVRGLTGPIGPPGPAGAPGDKGETGPSGPAGPTGARGAPGDRGEPGPPGPAGFAGPPGADGQPGAKGEP
GDAGAKGDAGPPGPAGPAGPPGPIGNVGAPGPKGARGSAGPPGATGFPGAAGRVGPPGPSGNAGPPGPPGPAGK
E
GSKGPRGETGPAGRPGEVGPPGPPGPAGEKGAPGADGPAGAPGTPGPQGIAGQRGVVGLPGQRGERGFPGLPGPS
GEPGKQGPSGASGERGPPGPMGPPGLAGPPGESGREGAPGAEGSPGRDGAPGAKGDRGETGPAGPPGAPGAPGA
P
GPVGPAGKSGDRGETGPAGPAGPIGPVGARGPAGPQGPRGDKGETGEQGDRGIKGHRGFSGLQGPPGPPGSPGEQ
GPSGASGPAGPRGPPGSAGTPGKDGLNGLPGPIGPPGPRGRTGDAGPAGPPGPPGPPGPPGPPSGGYDLSFLPQPP
Q EKAHDGGRYYRA

>Preliminary_Springbok_A_COL1A2

QFDGKGGGPGPMGLMGPRGPPGASGAPGPQGFQGPPGEPGEPGQTGPAGARGPPGPPGKAGEDGHPGKPGRPGE
RGVVGPQGARGFPGTPGLPGFKGIRGHNGLDGLKGQPGAPGVKGEPGAPGENGTPGQTGARGLPGERGRVGAPG
PAGARGSDGSVGPVGPAGPIGSAGPPGFPGAPGPKGELGPVGNPGPAGPAGPRGEVGLPGLSGPVGPPGNPGANG
LPGAKGAAGLPGVAGAPGLPGPRGIPGPVGAAGATGARGLVGEPGPAGSKGESGNKGEPGAVGQPGPPGPSGEE
GKRGSTGEIGPAGPPGPPGLRGNPGSRGLPGADGRAGVMGPAGSRGATGPAGVRGPNGDSGRPGEPGLMGPRGF
PGSPGNIGPAGKEGPVGLPGIDGRPGPIGPAGARGEPGNIGFPGPKGPTGDPGKAGEKGHAGLAGPRGAPGPDGN
N
GAQGPPGLQGVQGGKGEQGPAGPPGFQGLPGPAGTAGEAGKPGERGIPGEFGLPGPAGARGERGPPGESGAAGP
AGPIGSRGPSGPPGPDGNKGEPGVVGAPGTAGPSGPSGLPGERGAAGIPGGKGEKGETGLRGDVGSPGRDGARGA
PGAVGAPGPAGANGDRGEAGAAGPAGPAGPRGSPGERGEVGPAGPNGFAGPAGAAGQPGAKGERGTKGPKGE
N
GPVGPTGPAGAAGPSGPNGPPGPAGSRGDGGPPGATGFPGAAGRTGPPGPAGISGPPGPPGPAGKEGLRGPRGDQ
GPVGRTGETGASGPPGFAGEKGPSGEPGTAGPPGTPGPQGLLGAPGFLGLPGSRGERGLPGVAGSVGEPGPLGIAG
PPGARGPPGNVGNPGVNGAPGEAGRDGNPGNDGPPGRDGQPGHKGERGYPGNAGPVGAVGAPGPQGPVGPAG
K
HGNRGEPGPAGAVGPAGAVGPRGPSGPQGIRGDKGEPGDKGPRGLPGLKGHNGLQGLPGLAGHHGDQGAPGAV
GPAGPRGPAGPTGPAGKDGRTGQPGAVGPAGIRGSQGSQGPAGPPGPPGPPGPPGPSGGGYDFGFDGDFYRA

b. ***Final predicted collagen 1 alpha 1 and 2 sequences for springbok* (Antidorcas marsupialis)**.

X indicates coverage in modern springbok sample (A) was not secure enough to be assigned (not detected, or detected only once). In addition, all potential isoleucines (I) from the original sheep sequence have been presented as leucines (L) for consistency as they cannot be distinguished with this type of mass spectrometry. Wild Bovid ZooMS marker m/z 1532 is highlighted in COL1A2.

>Springbok_COL1A1_ItoL

XLSYGYDEKSTGLSVPGPMGPSGPRGLPGPPGAPGPQGFQGPPGEPGEPGASGXXGPRGPPGPPGKNGDDGEAGK
PGRPGERGPPGPQGARGLPGTAGLXGXXGXRGFSGLDGAKGDAGPAGPKGEPGSPGENGAPGQMGPRGLPGER
G
RPGAPGPAGARGXXGXTGAAGPPGPTGPAGPPGFPGAVGAKGEAGPQGPRGSEGPQGVRGEPGPPGPAGAAGPA
GNPGADGQPGAKGANGAPGLAGAPGFPGARGPSGPQGPSGPPGPKGNSGEPGAPGSKGDTGAKGEPGPTGLQGP
PGPAGEEGKRGARGEPGPAGLPGPPGERGGPGSRGFPGADGVAGPKGPAGERGAPGPAGPKGSPGEAGRPGEAG
LPGAKGLTGSPGSPGPDGKTGPPGPAGQDGRPGPPGPPGARGQAGVMGFPGPKGAAGEPGKAGERGVPGPPGAV
GPAGKDGEAGAQGPPGPAGPAGERGEQGPAGSPGFQGLPGPAGPPGEAGKPGEQGVPGDLGAPGPSGARGERGF
PGERGVQGPPGPAGPRGAXGAPGNDGAKGDAGAPGAPGSQGAPGLQGMPGERGAAGLPGPKGDRGDAGPKGA
DGAPGKDGVRGLTGPLGPPGPAGAPGDKGETGPSGPAGPTGARGAPGDRGEPGPPGPAGFAGPPGADGQPGAKG
EPGDAGAKGDAGPPGPAGPAGPPGPLGNVGAPGPKGARGSAGPPGATGFPGAAGRVGPPGPSGNAGPPGPPGPA
GKEGSKGPRGETGPAGRPGEVGPPGPPGPAGEKGAPGADGPAGAPGTPGPQGLAGQRGVVGLPGQRGERGFPGL
PGPSGEPGKQGPSGASGERGPPGPMGPPGLAGPPGESGREGAPGAEGSPGRDGAPGAKGDRGETGPAGPPGAPGA
PGAPGPVGXXGKSGDRGETGPAGPAGPLGPVGARGPAGPQGPRGDKGETGEQGDRGLKGHRGFSGLQGPPGPPG
SPGEQGPSGASGPAGPRGPPGSAGTPGKDGLNGLPGPLGPPGPRGRTGDAGPAGPPGPPGPPGPPGPPSGGYDLSFL
PQPPQEKXXXXXXXXXXX

>Springbok_COL1A2_ItoL

XXXXXGGGPGPMGLMGPRGPPGASGAPGPQGFQGPPGEPGEPGQTGXXGARGPPGPPGKAGEDGHPGKPGRPG
E
RGVXGXXGXRGFPGTPGLPGFKGLXXXXGXXGXXGXXGXXGXXGEPGAPGEXGTPGQTGXXGXXGXXGRVG
APGPAGARGSDGSVGPVGPAGPLGSAGPPGFPGAPGPKGELGPVGNPGPAGPAGPRGEVGLPGLSGPVGPPGNPG
ANGLPGAXGAAGLPGVAGAPGLPGPRGLPGPVGAAGATGARGLVGEPGPAGSKGESGNKGEPGAVGQPGPPGPS
GEEGKRGSTGELGPAGPPGPPGLRGNPGSRGXXGXXGXXGVMGPAGSRGXXGXXGVRGPNGDSGRPGEPGLMG
PRGFPGSPGNLGPAGKEGPVGLPGLDGRPGPLGPAGARGEPGNLGFPGPKGPTGDPGKAGEKGHAGLAGPRGAP

GPDGNNGAQGPPGLQGVQGGKGEQGPAGPPGFQGLPGPAGTAGEAGKPGERGLPGEFGLPGPAGARGERGPPGE
SGAAGPAGPLGSRGPSGPPGPDGNKGEPGVVGAPGTAGPSGPSGLPGERGAAGLPGGKGEKGETGLRGDVGSPG
RDGARGAPGAVGAPGPAGAXGDRGEAGAAGPAGPAGPRGSPGERGEVGPAGPNGFAGPAGAAGQPGAKGXXG
TKGPKGENGPVGPTGPAGAAGPSGPNGPPGPAGSRGDGGPPGATGFPGAAGRTGPPGPAGLSGPPGPPGPAGKEG
LRGPRGDQGPVGRTGXXGXSGPPGFAGEKGPSGEPGTAGXXGXXGXXGXLGAPGFLGLPGSRGERGLPGVAGS
V
GEPGPLGLAGPPGARGPPGNVGNPGVNGAPGEAGRDGNPGNDGPPGRDGQPGHKGERGYPGNAGPVGAVGAPG
PQGPVGPAGKXGXX**GEPGPAGAVGPAGAVGPR**GPSGPQGLRGDXGXXGXXGXRGXXGXXGXXGXXGLPGLA
GHHGDQGAPGAVGPAGPRGPAGPTGPAGKDGRTGQPGAVGPAGLRGSQGSQGPAGPPGPPGPPGPPGPSGGGYD
FGFDGDFYR

6. Spoegrivier P4859C Sheep Proteome

After the archaeological sample P4859C from Spoegrivier was determined to be sheep, a MaxQuant search for all proteins present was performed to detect other potential sheep specific proteins, as well as investigate the high recovery of archived collagen samples extracted for radiocarbon dating analysis. Species specificity is based only on sequences publicly available in the NCBI database, and we acknowledge that there could be matches to other species for which the sequence is not available.

Table S3: Recovered Proteome of Archaeological Sheep Sample Spoegrivier P4859C
Combined results of MaxQuant semi-tryptic and semi-elastase searches for archaeological sample Spoegrivier P4859C against sheep proteome (Uniprot). If a peptide was found in both searches, it was only counted once, as well as the matched spectra for that peptide. Proteins with blue backgrounds were found only in the tryptic search, green backgrounds were discovered in both trypsin and elastase searches, and the one in orange was found in the elastase search exclusively. Total sequence coverage percentage between both searches was calculated using Protein Coverage Summarizer (Pacific Northwest National Laboratory, https://omics.pnl.gov/software/protein-coverage-summarizer). Closest Taxonomic hit was determined from the specificity of NCBI's pBLAST searches of the peptides recovered.

Protein Name	Closest Taxonomic hit	Razor + Unique peptides	Unique peptides	Total Sequence Coverage (%)	Sequence length	Matched spectra
Collagen type I alpha 1 chain	*Ovis aries*	729	697	69.2	1474	2209
Collagen type I alpha 2 chain	*Ovis aries*	603	603	74.6	1364	1512
Alpha-2-HS-glycoprotein	*Ovis aries*	23	23	26.1	364	62
Chondroadherin	Pecora	18	18	44.1	358	50
SERPIN domain-containing protein (Pigment Epithelium Derived Factor)	Bovidae, Phyllostomus discolor, Elephantulus edwardii	15	15	35.3	416	31
Collagen type V alpha 2 chain	Pecora	17	17	14.5	1499	25

Collagen type V alpha 1 chain	*Ovis aries*	13	10	11.2	1830	27
Biglycan	Eutheria	12	12	24.4	369	44
Lumican	Pecora	7	7	14.6	342	12
Kazal-like domain-containing protein (Osteonectin)	Amniota	6	6	11.3	462	8

Collagen type II alpha 1 chain	Amniota	8	7	4.6	1490	50
SMB domain-containing protein (Vitronectin)	*Ovis aries*	5	5	13.9	475	6
Prothrombin (based on homology)	*Ovis aries*	4	4	6.8	666	9
Collagen type XI alpha 1 chain	Artiodactyla	4	4	4	1805	11
Osteocalcin	*Ovis aries*, *Lontra canadensis*, or *Enhydra lutris kenyoni*	3	3	14.7	129	6
Collagen type XII alpha 1 chain	Theria	3	3	1.4	3125	3
Osteomodulin	Pecora	3	3	7.9	420	11
Periostin	Eutheria	3	3	4.7	808	3
Vitamin K-Dependent Protein S	*Ovis aries* or *Capra hircus*	3	3	4.9	675	6
Collagen type VI alpha 3 chain	*Ovis aries* or *Capra hircus*	3	3	1.5	3154	3
Thrombospondin-1	Eutheria	2	2	2.1	1170	3
Osteoglycin	Laurasiatheria	2	2	8.4	299	7
Heparan sulfate proteoglycan 2 (Perlecan)	*Ovis aries* or *Capra hircus*	2	2	0.7	4260	2
Nucleobindin-1	Eutheria	2	2	5.2	460	4
Collagen type V alpha 3 chain	Boreoeutheria	2	2	1.6	1746	4

Table S4: Species Informative Peptides of Archaeological Sheep Sample Spoegrivier P4859C

Combined results of MaxQuant semi-tryptic and semi-elastase searches for archaeological sample Spoegrivier P4859C against sheep proteome (Uniprot). In some cases, specific peptides alone are not indicative of a genus, so the overlap between identifications of more than one peptide determines the genus. MaxQuant score refers to the best matched spectrum.

Protein name	Sequences used for identification	BLAST ID	Length (aa)	Mass	Charge	MaxQuant Score	Matched Spectra
Collagen type I alpha 1 chain	AGEVGPPGPPGPA	*Ovis aries* and various Cetacea	13	1101.5455	2	152.63	2
	AGEVGPPGPPGPAG	*Ovis aries* and various Cetacea	14	1158.5669	2	141.2	1
	AGEVGPPGPPGPAGE	*Ovis aries* and various Cetacea	15	1287.6095	2	335.66	1
	AGEVGPPGPPGPAGEK	*Ovis aries* and various Cetacea	16	1415.7045	2;3	250.13	9
	AGEVGPPGPPGPAGEKG	*Ovis aries* and various Cetacea	17	1472.726	2	120.23	1
	AGEVGPPGPPGPAGEKGA	*Ovis aries* and various Cetacea	18	1543.7631	2	159.77	1
	AGEVGPPGPPGPAGEKGA PG	*Ovis aries* and various Cetacea	20	1697.8373	2	206.55	4
	AGEVGPPGPPGPAGEKG A PGA	*Ovis aries* and various Cetacea	21	1768.8744	2	237.1	3
	AGEVGPPGPPGPAGEK GA PGAD	*Ovis aries* and various Cetacea	22	1883.9014	2;3	279.19	10
	AGEVGPPGPPGPAGEK GA PGADGPA	*Ovis aries* and various Cetacea	25	2109.0127	2;3	400.27	8
	EGAPGAEGSPGRDGAP GA KGD	*Ovis aries, Capra hircus, Desmodus rotundus, Phyllostomus discolor*	21	1851.834 7	3	123.19	1
	GRAGEVGPPGPPGPA	*Ovis aries* and various Cetacea	15	1314.668	2	159.83	1

	GRAGEVGPPGPPGPAGE K	*Ovis aries* and various Cetacea	18	1628.827 1	2	255.96	1
	GRAGEVGPPGPPGPAGEK GAPGAD	*Ovis aries* and various Cetacea	24	2097.0239	2;3	276.05	6
	AAGPPGFVGEKGPSGE PG TAGPPG	*Ovis aries* and *Capra hircus*	24	2090.006 9	2	231.37	1
Collagen type I alpha 2 chain	AGPPGTPGPQGLLGAP GFL GLPG	*Ovis aries*, *Camelus* sp., *Sus* sp., *Bos* sp., *Vicugna* sp., *Muntiacus* sp.	23	2027.084	2	186.13	1
	AGPPGTPGPQGLLGAP GFL GLPGSR	*Ovis aries*, *Camelus* sp., *Sus* sp., *Bos* sp., *Vicugna* sp., *Muntiacus* sp.	25	2270.217 2	2;3	267.39	3
	AGPVGAAGAPGPQGPV GP TGK	*Ovis aries* and *Capra hircus*	21	1741.911 1	2;3	196.18	3
	EPGAAGPPGFVGEK	*Ovis aries* and *Capra hircus*	14	1311.6459	2	113.22	1
	EPGPVGAVGPAGAVGPR	*Ovis aries* and *Capra hircus*	17	1486.7892	2	122.18	2
	GEPGPVGAVGPAGAVGP R	*Ovis aries* and *Capra hircus*	18	1543.8107	2;3	248.19	7
	GEPGTAGPPGTPGPQGLL	various but not *Capra hircus*	18	1601.8049	2	154.74	1
	GFPGSPGNIGPAGKEGPA	*Ovis aries* and *Capra hircus*	18	1608.7896	2	133	2
	GLLGAPGFLGLPGSR	various but not *Capra hircus*	18	1752.9635	3	132.47	1
	GLLGAPGFLGLPGSRGE R	various but not *Capra hircus*	24	2199.18	2;3	365.68	9
	GNAGPVGAAGAPGPQGP V GPTGK	*Ovis aries* and *Capra hircus*	23	1912.975 5	2	117.18	1
	GPAGPTGPAGKDGRT	*Ovis aries* and *Capra hircus*	15	1337.6688	2;3	232.48	2

	GPAGPTGPAGKDGRTGQP GAVGPA	*Ovis aries* and *Capra hircus*	24	2072.0399	3	149.93	3
	GPPGTPGPQGLL	various but not *Capra hircus*	12	1089.5819	2	184.04	1
	GPPGTPGPQGLLGAPGFL	*Ovis aries*, *Camelus* sp., *Sus* sp., *Bos* sp., *Vicugna* sp., *Muntiacus* sp.	18	1631.8671	2	168.22	1
	GPPGTPGPQGLLGAPGFLG L	*Ovis aries*, *Camelus* sp., *Sus* sp., *Bos* sp., *Vicugna* sp., *Muntiacus* sp.	20	1801.9727	2	176.78	1
	GPPGTPGPQGLLGAPGFLG LPG	*Ovis aries*, *Camelus* sp., *Sus* sp., *Bos* sp., *Vicugna* sp., *Muntiacus* sp.	22	1956.0469	2	164.53	1
	GPPGTPGPQGLLGAPGFLG LPGSR	*Ovis aries*, *Camelus* sp., *Sus* sp., *Bos* sp., *Vicugna* sp., *Muntiacus* sp.	15	1410.7983	2	162.38	2
	GPQGLLGAPGFLGLPGSR	various but not *Capra hircus*	18	1692.9311	2	232.5	3
	GPQGLLGAPGFLGLPGSR GER	various but not *Capra hircus*	21	2035.0963	3	191.95	3
	GPSGEPGTAGPPGTPGPQG L	various but not *Capra hircus*	20	1729.8271	2	236.28	1
	GPSGEPGTAGPPGTPGPQG LL	various but not *Capra hircus*	21	1842.9112	2	278.84	2
	GPSGEPGTAGPPGTPGPQG LLG	various but not *Capra hircus*	22	1899.9327	2	230.69	2
	GPSGEPGTAGPPGTPGPQG LLGA	various but not *Capra hircus*	23	1970.9698	2	225.65	1

	GPSGEPGTAGPPGTPGP QG LLGAPG	various but not *Capra hircus*	25	2125.044	2;3	285.81	4
	GPVGAAGAPGPQGPV GPT GK	*Ovis aries* and *Capra hircus*	20	1670.874	2	207.31	2
	GPVGAVGPAGAVGPR	*Ovis aries* and *Capra hircus*	15	1260.6939	2	242.08	1
	GTPGPQGLLGAPGFLGL PG SR	various but not *Capra hircus*	21	1948.053	2;3	126.61	5
	GYPGNAGPVGAAGAP GPQ GPVGPTG	*Ovis aries* and *Capra hircus*	25	2102.018 1	2	227.29	2
	HGSRGEPGPVGAVGPA GA VGPR	*Ovis aries* and *Capra hircus*	22	1981.0242	3;4	153.19	2
	LGAPGFLGLPGSR	various but not *Capra hircus*	13	1240.6928	2	145.23	2
	LGAPGFLGLPGSRGER	various but not *Capra hircus*	16	1582.858	2;3	172.13	2
	LLGAPGFLGLPGSR	various but not *Capra hircus*	14	1353.7769	2	164.43	3
	LLGAPGFLGLPGSRGER	various but not *Capra hircus*	17	1695.942	3	125.52	2
	NAGPVGAAGAPGPQGP VG PTGK	*Ovis aries* and *Capra hircus*	22	1855.9541	2	200.96	5
	PGNAGPVGAAGAPGPQ GP VGPTGK	*Ovis aries* and *Capra hircus*	24	2010.028 3	2;3	329.21	5
	PGPQGLLGAPGFLGLPGSR	various but not *Capra hircus*	19	1789.9839	2;3	318.46	6
	PGPVGAVGPAGAVGPR	*Ovis aries* and *Capra hircus*	16	1357.7466	2	259.4	2
	PGTAGPPGTPGPQGLL	various but not *Capra hircus*	16	1415.7409	2	180.66	1
	PGTPGPQGLLGAPGFL GLP GSR	various but not *Capra hircus*	22	2045.105 8	2;3	289.1	4

	PPGTPGPQGLLGAPGFL GL PGSR	various but not *Capra hircus*	23	2142.158 6	2;3	184.22	5
	PQGLLGAPGFLGLPGSR	various but not *Capra hircus*	17	1635.9097	2;3	230.23	4
	PQGLLGAPGFLGLPGSR GE R	various but not *Capra hircus*	20	1978.0748	3	186.23	1
	PVGAAGAPGPQGPVGP TG K	*Ovis aries* and *Capra hircus*	19	1613.8526	2;3	215.87	3
	QGLLGAPGFLGLPGSR	various but not *Capra hircus*	16	1538.8569	2	107.65	2
	RGEPGPVGAVGPAGAV GP R	*Ovis aries* and *Capra hircus*	19	1699.9118	2;3	107.75	2
	TAGPPGTPGPQGL	various but not *Capra hircus*	13	1148.5826	2	191.4	1
	TAGPPGTPGPQGLL	various but not *Capra hircus*	14	1261.6667	2	205.32	2
	TGEPGAAGPPGFVG	*Ovis aries* and *Capra hircus*	14	1212.5775	2	131.22	1
	TGEPGAAGPPGFVGE	*Ovis aries* and *Capra hircus*	15	1341.6201	2	197.79	1
	TGEPGAAGPPGFVGEK	*Ovis aries* and *Capra hircus*	16	1469.7151	2	175.48	5
	TGEPGAAGPPGFVGEKG	*Ovis aries* and *Capra hircus*	17	1526.7365	2	124.48	1
	TGEPGAAGPPGFVGEK GP S	*Ovis aries* and *Capra hircus*	19	1710.8213	2	196.37	1
	TGEPGAAGPPGFVGEK GP SGEPG	*Ovis aries* and *Capra hircus*	23	2050.959 6	2	236.26	1
	TGEPGAAGPPGFVGEK GP SGEPGT	*Ovis aries* and *Capra hircus*	24	2152.007 3	2	227.41	1
	TGEPGAAGPPGFVGEKGP SGEPGTA	*Ovis aries* and *Capra hircus*	25	2223.044 4	2	248.75	1

	TPGPQGLLGAPGFLGLPG S R	various but not *Capra hircus*	20	1891.0316	2;3	302.4	6
	TPGPQGLLGAPGFLGLP GS RGER	various but not *Capra hircus*	23	2233.1967	3	181.75	2
	VGAVGPAGAVGPR	*Ovis aries* and *Capra hircus*	13	1106.619 6	2	116.37	1
	AAGLPVGSVVAGPSVVA V PLPLHR	*Ovis aries*	24	2262.3212	3	119.16	1
Alpha-2-HS glycoprotein	AQFVPLPGSVSVEF	*Ovis aries*	14	1475.766	2	164.33	2
	AQFVPLPGSVSVEFA	*Ovis aries*	15	1546.803 1	2	93.561	1
	GLPVGSVVAGPSVVAVP L PLHR	*Ovis aries*	22	2120.247	3	114.72	1
	LPVGSVVAGPSVVAVP LP LHR	*Ovis aries*	21	2063.225 5	3	179.46	1
	IAQLPLTGSTSIIFFLPQ K	Bovidae, *Phyllostomus discolor*, *Elephantulus edwardii*	19	2073.187 4	2;3	162.37	5
SERPIN domain containing protein (Pigment Epithelium Derived Factor)	TGSTSIIFFLPQK	Bovidae, *Phyllostomus discolor*, *Elephantulus edwardii*	13	1437.786 8	2	103.22	1
	PGPSGPPGPPGEDGERGD D GEAGPR	*Ovis aries*	25	2356.0316	3	184.59	2
Collagen type V alpha 1 chain	FEDGVLEPEFPR	*Ovis aries*	12	1433.682 7	2	167.09	2
SMB domain containing protein (Vitronectin)	LQDKTEAELFESYIEGR	*Ovis aries*	17	2026.9848	3	132.51	1
Prothrom bin (based on homology)	TEAELFESYIEGR	*Ovis aries*	13	1542.720 2	2;3	219.03	3
	IEEGVPQLLIVLTADR	*Ovis aries*, *Capra hircus*, *Muntiacus* sp., *Echinops telfairi*	16	1764.9986	2	120.87	1

Collagen type VI alpha 3 chain	LLTPITTLTAGQIQQLLAS T R	Bovidae	21	2238.2947	3	136.46	1
	NNLELLTQLR	*Ovis aries* and *Capra hircus*	10	1212.682 6	2	164.68	3
Vitamin K Dependent Protein S	PGLGAPAPYPDPLEPR	*Ovis aries, Lontra canadensis, Enhydra lutris kenyoni*	16	1645.8464	2	172.92	2
Osteocalcin	YLDPGLGAPAPYPDPLEP R	*Ovis aries, Lontra canadensis, Enhydra lutris kenyoni*	19	2037.0207	2;3	104.89	2
Heparan sulfate proteo glycan 2 (Perlecan)	VVVGSVPLESSVLVR	*Ovis aries* and *Capra hircus*	15	1538.9032	2	117.25	1

7. Re-analysis of Le Meillour *et al.* (2020) springbok samples

Figure S3: *Coverage of the GEPGPAGAVGPAGAVGPR wild bovid marker in Le Meillour* et al. *(2020) springbok samples a)SPR89 and b)SPR1670. Searches performed with PEAKS software based on original reference parameters (without phosphorylation), against the above springbok collagen 1 sequence, as well as the sheep, goat, and cow versions.*

a.) Wild bovid marker *m/z* 1532 found in Le Meillour *et al.* (2020) SPR89

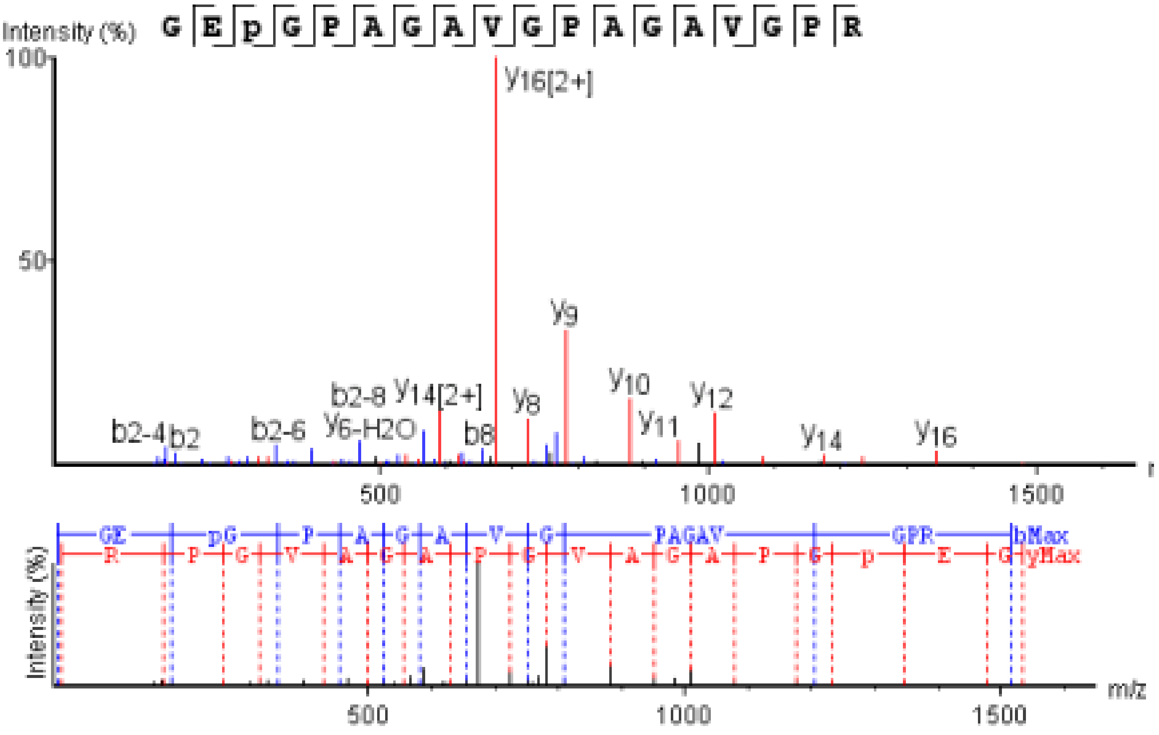

b.) Wild bovid marker *m/z* 1532 found in Le Meillour *et al.* (2020) SPR1670

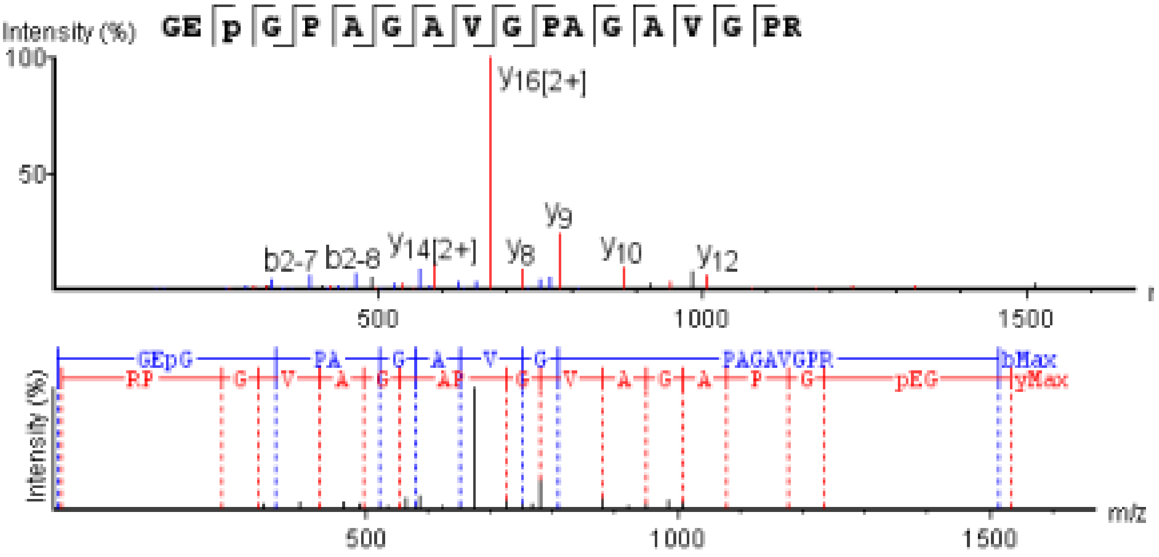

8. Supplementary References

1. Fiddyment, S. *et al.* Animal origin of 13th-century uterine vellum revealed using noninvasive peptide fingerprinting. *Proceedings of the National Academy of Sciences* **112**, 15066–15071 (2015).
2. McGrath, K. *et al.* Identifying Archaeological Bone via Non-Destructive ZooMS and the Materiality of Symbolic Expression: Examples from Iroquoian Bone Points. *Nature Scientific Reports* **9**, 11027 (2019).
3. Kirby, D. P., Manick, A. & Newman, R. Minimally Invasive Sampling of Surface Coatings for Protein Identification by Peptide Mass Fingerprinting: A Case Study with Photographs. *Journal of the American Institute for Conservation* (2019) doi:10.1080/01971360.2019.1656446.
4. Brown, Samantha, Katerina Douka, Matthew Collins, and Kristine Korzow Richter. "On the Standardization of ZooMS Nomenclature." *Journal of Proteomics* (2020) doi:10.1016/j.jprot.2020.
5. Strohalm, M., Kavan, D., Novák, P., Volný, M. & Havlícek, V. mMass 3: a cross-platform software environment for precise analysis of mass spectrometric data. *Analytical Chemistry* **82**, 4648–4651 (2010).
6. Jensen, T. Z. T. *et al.* The biomolecular characterization of a finger ring contextually dated to the emergence of the Early Neolithic from Syltholm, Denmark. *Royal Society Open Science* **7**, 191172 (2020).
7. Mackie, M. *et al.* Palaeoproteomic Profiling of Conservation Layers on a 14th Century Italian Wall Painting. *Angewandte Chemie International Edition* **57**, 7369–7374 (2018).
8. Cox, J. & Mann, M. MaxQuant enables high peptide identification rates, individualized ppb-range mass accuracies and proteome-wide protein quantification. *Nature Biotechnology* **26**, 1367–1372 (2008).
9. Han, X., He, L., Xin, L., Shan, B. & Ma, B. PeaksPTM: Mass spectrometry-based identification of peptides with unspecified modifications. *Journal of Proteome Research* **10**, 2930–2936 (2011).
10. Shoulders, M. D. & Raines, R. T. Collagen structure and stability. *Annual Review of Biochemistry* **78**, 929–958 (2009).
11. Katoh, K., Rozewicki, J. & Yamada, K. D. MAFFT online service: multiple sequence alignment, interactive sequence choice and visualization. *Briefings in Bioinformatics* **20**, 1160–1166 (2019).

12. Cappellini, E. *et al.* Early Pleistocene enamel proteome from Dmanisi resolves Stephanorhinus phylogeny. *Nature* **574**, 103–107 (2019).
13. Buckley, M. *et al.* Distinguishing between Archaeological Sheep and Goat Bones Using a Single Collagen Peptide. *Journal of Archaeological Science* **37**, 13-20 (2010).
